# Effects of Copper Exposure on Oxidative Stress, Apoptosis, Endoplasmic Reticulum Stress, Autophagy and Immune Response in Different Tissues of Chinese Mitten Crab (*Eriocheir sinensis*)

**DOI:** 10.3390/antiox11102029

**Published:** 2022-10-14

**Authors:** Wenrong Feng, Shengyan Su, Changyou Song, Fan Yu, Jun Zhou, Jianlin Li, Rui Jia, Pao Xu, Yongkai Tang

**Affiliations:** 1 Key Laboratory of Freshwater Fisheries and Germplasm Resources Utilization, Ministry of Agriculture and Rural Affairs, Freshwater Fisheries Research Center, Chinese Academy of Fishery Sciences, Wuxi 214081, China; 2 Freshwater Fisheries Research Institute of Jiangsu Province, Nanjing 210017, China

**Keywords:** copper, apoptosis, endoplasmic reticulum stress, oxidative stress, *Eriocheir sinensis*

## Abstract

High concentrations of copper (Cu^2+^) pose a great threat to aquatic animals. However, the mechanisms underlying the response of crustaceans to Cu^2+^ exposure have not been well studied. Therefore, we investigated the alterations of physiological and molecular parameters in Chinese mitten crab (*Eriocheir sinensis*) after Cu^2+^ exposure. The crabs were exposed to 0 (control), 0.04, 0.18, and 0.70 mg/L of Cu^2+^ for 5 days, and the hemolymph, hepatopancreas, gills, and muscle were sampled. The results showed that Cu^2+^ exposure decreased the antioxidative capacity and promoted lipid peroxidation in different tissues. Apoptosis was induced by Cu^2+^ exposure, and this activation was associated with the mitochondrial and ERK pathways in the hepatopancreas. ER stress-related genes were upregulated in the hepatopancreas but downregulated in the gills at higher doses of Cu^2+^. Autophagy was considerably influenced by Cu^2+^ exposure, as evidenced by the upregulation of autophagy-related genes in the hepatopancreas and gills. Cu^2+^ exposure also caused an immune response in different tissues, especially the hepatopancreas, where the TLR2-MyD88-NF-κB pathway was initiated to mediate the inflammatory response. Overall, our results suggest that Cu^2+^ exposure induces oxidative stress, ER stress, apoptosis, autophagy, and immune response in *E. sinensis*, and the toxicity may be implicated following the activation of the ERK, AMPK, and TLR2-MyD88-NF-κB pathways.

## 1. Introduction

Copper (Cu) is an essential metal element for all living organisms and mainly exists in Cu^2+^ and Cu^+^ states. It is involved in a variety of physiological functions, such as electron transport, mitochondrial function, and free radical scavenging [1]. When present in excess, however, Cu becomes toxic and causes damage to cellular components [2]. It is also classified as a priority environmental pollutant [3]. Cu can accumulate in aquatic systems from both natural (e.g., erosion of rocks and soils, geological deposition) and anthropogenic sources (e.g., industrial, mining and agricultural activities, sewage discharge) [3,4]. In aquaculture, copper sulfate (CuSO_4_) has been extensively used as a therapeutic agent to control skin lesions and gill diseases caused by parasites and pathogenic bacteria [5]. It is further used globally as an algicide to control harmful cyanobacterial blooms in freshwater [6]. The extensive use of Cu^2+^ may lead to its short-term and/or repeated accumulation in aquatic environments. High concentrations of Cu^2+^ (up to 100 mg/L) have further been detected in various aquatic ecosystems [7].

High concentrations of copper have been reported to be potentially toxic to aquatic animals in aquatic environment [8]. Liver and gills are the primary sites of Cu toxicity in freshwater fish, where accumulated Cu^2+^ disrupts regular Cu homeostasis and branchial ion regulation [9]. The tolerance to waterborne Cu^2+^ varies among aquatic animals, for example, 48 h LD_50_ 0.75 mg/L in *Oncorhynchus mykiss* [10] and 72 h LD_50_ 40.6 mg/L in *Oreochromis niloticus* [11]. In crustaceans, the safe concentration of Cu^2+^ is also variable, e.g., 0.02 mg/L in juvenile *Macrobrachium rosenbergii* [12], 0.375 mg/L in juvenile *procambarus clarkia* [13], and 0.008 mg/L in larval *Penaeus vannamei* [14]. Acute exposure (24–96 h) to Cu^2+^ (0.1–84.9 μM) decreases the rate of oxygen consumption and alters the swimming performance of fish [15,16]. Cu^2+^ also suppresses immune function by decreasing blood leukocytes in fish [17,18]. The toxicity of Cu^2+^ to crustaceans has also garnered attention. Long-term exposure to Cu^2+^ (0.1641 ppm, 30 days) suppresses the glutathione system in *Penaeus indicus* [19]. Furthermore, sub-lethal Cu^2+^ exposure leads to necrosis and the loss of regular structures in the gills and hepatopancreas of *Litopenaeus vannamei* [20]. However, the underlying mechanisms of Cu toxicity in crustaceans are not yet well understood; therefore, it is necessary to systematically examine the effects of Cu^2+^ exposure on crustaceans and its potential ecological risks in aquatic environments.

The Chinese mitten crab (*Eriocheir sinensis*) is one of the most commercially cultured aquatic species in China. When managing crab ponds, CuSO_4_ is commonly used to eradicate filamentous algae and control parasites and pathogens, which may lead to Cu^2+^ accumulation in the ponds. High ambient Cu^2+^ is a significant threat to the health of *E. sinensis.* After 24 h of Cu^2+^ exposure, the metabolism and osmotic regulation in the gills of *E. sinensis* are altered [21], and after 96 h, its molting, growth, and survival are suppressed [22,23]. Although Cu^2+^ toxicity in *E. sinensis* has garnered much attention in recent years, the knowledge of its underlying molecular mechanisms remains limited. It is unclear whether endoplasmic reticulum (ER) stress and autophagy are involved in Cu^2+^ toxicity in *E. sinensis*; the key signaling pathways have rarely been evaluated in Cu^2+^-induced inflammatory responses and apoptosis. In addition, it is important to determine whether there is tissue specificity in the response to Cu^2+^ toxicity.

In this study, we investigated the physiological and molecular responses of *E. sinensis* to Cu^2+^ and evaluated the potential molecular mechanisms. To this end, we exposed *E. sinensis* to different concentrations of Cu^2+^ for 5 days [24] and observed the changes in the redox state, apoptosis, ER stress, autophagy, immune response, and detoxification in different tissues. We also analyzed multiple key signaling pathways, including the inositol-requiring enzyme 1 (IRE1), mitogen-activated protein kinases (MAPKs), AMP-activated protein kinase (AMPK), and Toll-like receptor (TLR) pathways. Our findings provide new insights into the mechanisms underlying the toxicity of Cu^2+^ exposure in *E. sinensis*, which may contribute to the risk assessments of Cu^2+^ in aquatic environments.

## 2. Materials and Methods

### 2.1. Crab Rearing, Experimental Design, and Sample Collection

Healthy *E. sinensis* (120 ± 1.2 g) were obtained from the Freshwater Fisheries Research Center (Wuxi, China). The crabs were kept in indoor glass tanks (100 × 60 × 40 cm) for 7 days to acclimatize to the laboratory conditions (temperature, 25 ± 1 °C; dissolved oxygen > 5.0 mg/L; ammonia nitrogen < 0.1 mg/L; pH 8.0 ± 0.5). After acclimatizing, the crabs were randomly distributed into four groups and exposed to 0 (control), 0.04, 0.18, and 0.70 mg/L of copper for 5 days. Each group contained 36 crabs (pooled male and female, 1:1), and the experiment was performed in triplicate. The sub-lethal copper concentrations were chosen according to our previous study, where the 96 h LC_50_ of Cu^2+^ was 5.63 mg/L [24]. The Cu^2+^ concentrations were prepared and adjusted by adding CuSO_4_ (Aladdin, Shanghai, China). During the experiment, the water was renewed every day, and the crabs were fed a commercial diet (crude protein 42.6%, crude lipid 8.0%, crude ash 16.2%; HIPORE Feed Co., Ltd., Taizhou, China) at 1% of their body weight daily to avoid the adverse effects caused by hunger.

After 5 days of exposure, eight crabs from each tank were sampled randomly, and the hemolymph, hepatopancreas, gills, and muscle were immediately collected after anesthetization with an ice bath. Tissues from four crabs were mixed into one sample (six samples in total). The hemolymph was centrifuged (4000× *g* for 10 min at 4 °C) to obtain the supernatant. All samples were stored at −80 °C for gene expression and biochemistry analyses. The use of the crabs in the experiment was approved by the Freshwater Fisheries Research Center, and all experimental procedures were performed according to the Animal Care Guidelines.

### 2.2. Biochemical Assay

The hepatopancreas, gills, and muscle were homogenized nine times (*v/w*) with ice-cold normal saline (0.86% NaCl). The homogenized mixture was centrifuged at 3600 rpm at 4 °C for 10 min to collect the supernatant, which was used for biochemical assays. The levels of glutathione (GSH), glutathione *S*-transferase (GST), superoxide dismutase (SOD), total antioxidant capacity (T-AOC), malondialdehyde (MDA), and total protein (TP) in the hemolymph, hepatopancreas, gills, and muscle were measured as described by Jia, et al. [25]. Commercial kits for GSH, GST, SOD, T-AOC, MDA, and TP were purchased from Nanjing Jiancheng Bioengineering Institute (Nanjing, China) and Beyotime Biotechnology (Nantong, China).

### 2.3. Quantitative Real-Time PCR Analysis

Total RNA from the hepatopancreas, gills, and muscle was isolated using the RNAiso Plus reagent (TaKaRa, Beijing, China) according to the manufacturer’s instructions. The quality and quantity of total RNA were evaluated using OD_260_, the ratio of OD_260_/OD_280_, and agarose gel electrophoresis. The isolated RNA was used to synthesize cDNA via reverse transcription PCR using the PrimeScript™ RT reagent (TaKaRa, No. RR047). In brief, the RNA (1 μg) was mixed with gDNA Eraser at 42 °C for 2 min to remove the genomic DNA. The mixture was then reacted with PrimeScript RT Enzyme Mix I (1 μL), RT Primer Mix (1 μL), 5× PrimeScript Buffer 2 (4 μL), and RNase-Free dH_2_O (4 μL) for 15 min at 37 °C and 5 s at 85 °C.

The mRNA levels of the target genes were measured by quantitative real-time PCR (qPCR) on a CFX96 Real-Time PCR instrument (Bio-Rad, Hercules, CA, USA). During the qPCR amplification process, cDNA (2 μL), TB Green Premix Ex Taq II (TaKaRa; 12.5 μL), forward and reverse specific primers (1 μL), and RNase-free water (8.5 μL) were mixed. The mixture was incubated for 30 s at 95 °C and subjected to 40 cycles at 95 °C for 5 s and 59–61 °C for 1 min. The expression of the target genes was analyzed using the 2^−ΔΔCq^ method [26]. The primers used are listed in Table S1. The ubiquitin-conjugating enzyme E2b (UBE) and β-actin genes were used as internal references to normalize the quantification cycle (Cq) values [27].

### 2.4. Integrated Biomarker Response Analysis

The integrated biomarker response (IBR) analysis for the oxidative stress parameters of different tissues was conducted using the method described by Sanchez, et al. [28]. The control group (without Cu^2+^) was used as the reference condition. The IBR_v2_ value per concentration is the sum of the absolute values of the biomarker deviation index (A). The reference deviation of each biomarker is represented by the A value. In the star plot, the values above and below zero reflect the induction and reduction of the biomarker, respectively.

### 2.5. Statistical Analysis

All statistical analyses were performed using SPSS 24.0 (SPSS, Chicago, IL, USA). The results are expressed as the mean ± standard error of the mean (SEM). The normal distribution and heterogeneity of variance were evaluated using the Shapiro–Wilk and Bartlett tests, respectively. For comparisons among different groups, a one-way analysis of variance (ANOVA) was performed, followed by an LSD post hoc test in cases of equal variance or the Kruskal–Wallis test for unequal variance. Differences were considered statistically significant at *p* < 0.05 among the different groups.

## 3. Results

### 3.1. Alterations in the Redox State 

**Figure 1:**
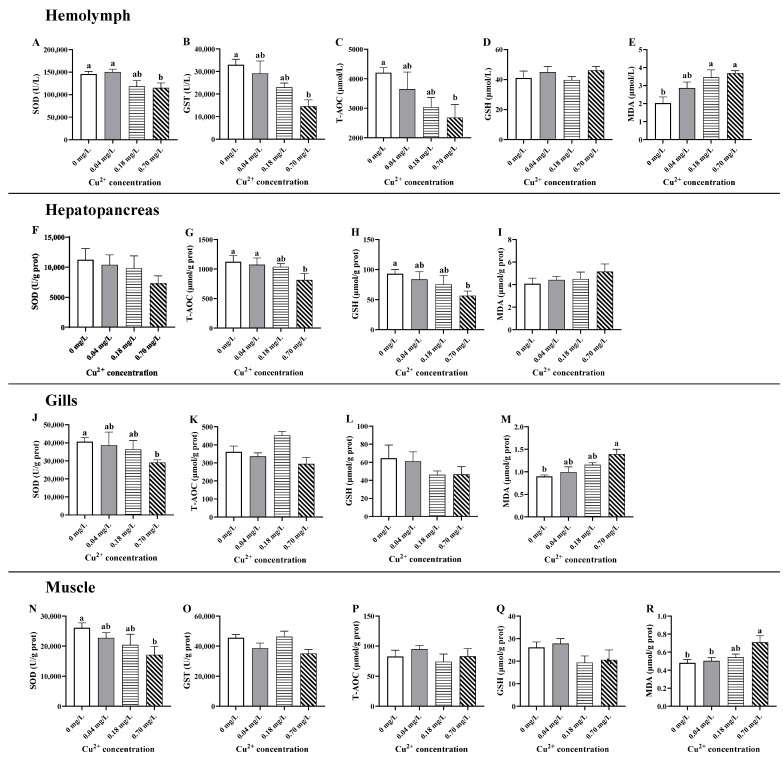
Changes in the oxidative stress parameters in different tissues of *E. sinensis* exposed to copper for 5 days. (**A**–**E**) hemolymph; (**F**–**I**) hepatopancreas; (**J**–**M**) gills; (**N**–**R**) muscle. The values are expressed the mean ± SEM (*n* = 6). Different letters denote significant differences among different groups (*p* < 0.05).

There was a linear decrease in the levels of T-AOC, SOD, and GST and an increase in MDA after treatment with different concentrations of Cu^2+^ in the hemolymph (Figure 1A–E). Compared to the control group, the decreases in T-AOC, SOD, and GST were statistically significant in the 0.70 mg/L Cu^2+^-exposed group (*p* < 0.05; Figure 1A–C), while the increase in MDA was statistically significant in the 0.18 and 0.70 mg/L Cu^2+^-exposed groups (*p* < 0.05; Figure 1D). GSH was not influenced by Cu^2+^ exposure in the hemolymph (*p >* 0.05; Figure 1E).

In the hepatopancreas, the level of T-AOC decreased with increasing Cu^2+^ concentrations, and the lowest value was observed after exposure to 0.70 mg/L of Cu^2+^ (*p* < 0.05; Figure 1G). Similarly, the level of GSH underwent a dose-dependent decrease, which was statistically significant in the 0.70 mg/L Cu^2+^-exposed group (*p* < 0.05; Figure 1H). SOD activity and MDA content did not exhibit significant alterations in the hepatopancreas among the different Cu^2+^-exposed groups (*p >* 0.05; Figure 1F,I).

In the gills, SOD activity showed a downward trend after Cu^2+^ exposure and was strongly decreased in crabs exposed to 0.70 mg/L of Cu^2+^ (*p* < 0.05; Figure 1J). Conversely, the MDA content exhibited a rising tendency and was enhanced in crabs exposed to 0.70 mg/L of Cu^2+^ (*p* < 0.05; Figure 1M). The levels of T-AOC and GSH showed a slight but non-significant alteration in the gills among the different groups (*p >* 0.05; Figure 1K,L).

In the muscle, exposure to 0.70 mg/L of Cu^2+^ markedly decreased SOD activity and enhanced MDA formation (*p* < 0.05; Figure 1N,R) but did not influence other parameters (*p >* 0.05; Figure 1O,P).

**Figure 2:**
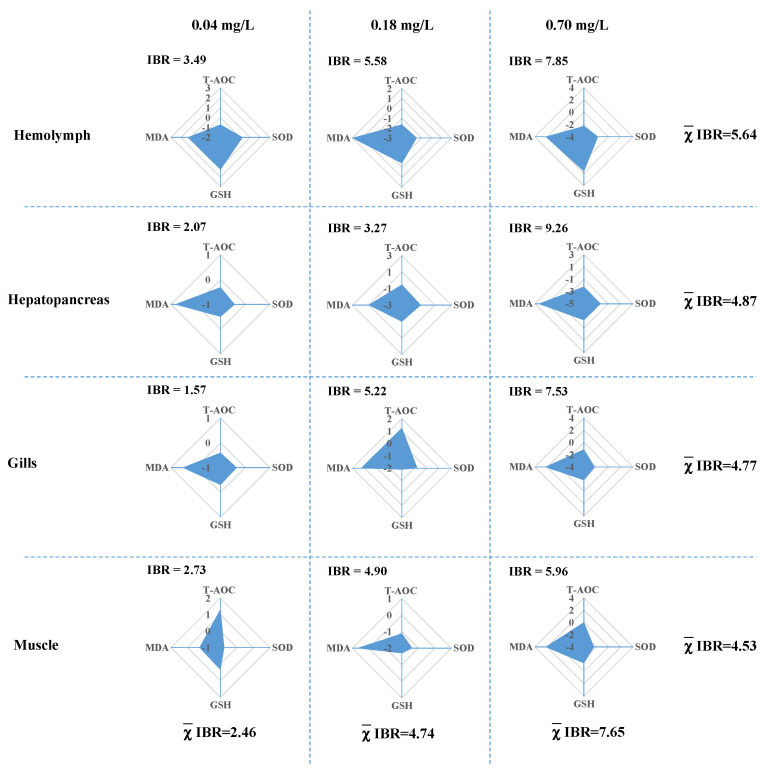
IBR index of the oxidative stress response to different concentrations of copper in the hemolymph, hepatopancreas, gills, and muscle. Biomarker values are represented in relation to the control group. The areas above zero reflect an increase in the biomarker, and the areas below zero reflect a decrease in the biomarker.

To compare the differences among the tissues and groups exposed to different concentrations of Cu^2+^, four biomarkers related to the redox state were standardized and depicted in a star plot (Figure 2). The IBRv2 index increased with increasing Cu^2+^ concentrations and exhibited dose-dependent toxicity. Among the different tissues, the following order of average IBR_v2_ values was observed: hemolymph (5.64) > hepatopancreas (4.87) > gills (4.77) > muscle (4.53). In addition, after exposure to 0.70 mg/L of Cu^2+^, the highest IBR_v2_ value was observed in the hepatopancreas (9.26).

### 3.2. Alterations in the Expression of Apoptosis-Related Genes

**Figure 3:**
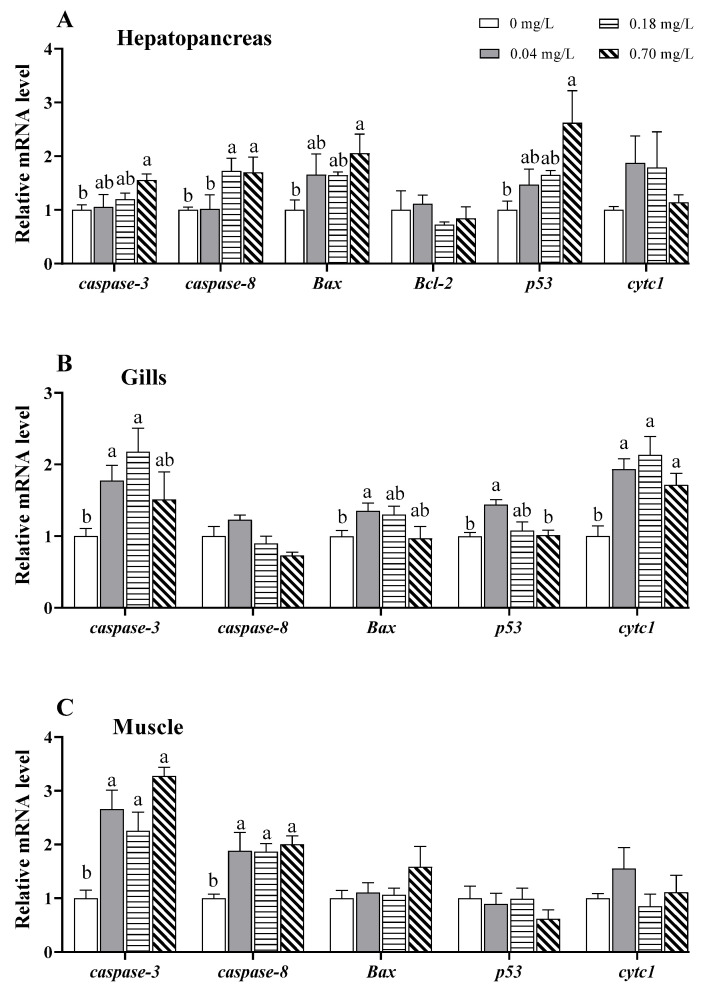
Expression of apoptosis-related genes in different tissues of *E. sinensis* exposed to copper for 5 days. (**A**) Hepatopancreas; (**B**) gills; (**C**) muscle. The values are expressed as the mean ± SEM (*n* = 6). Different letters denote significant differences among different groups (*p* < 0.05).

To evaluate whether Cu^2+^ exposure could induce apoptosis, we measured the mRNA levels of apoptosis-related genes, including *caspase-3*, *caspase-8*, B-cell lymphoma 2 (*Bcl-2*), Bcl2 X protein (*Bax*), *p53*, and cytochrome c (*cytc1*) in the hepatopancreas, gills, and muscle (Figure 3). In the hepatopancreas, the mRNA levels of *caspase-3*, *caspase-8*, *Bax*, and *p53* showed an increasing tendency, and the highest value of the expression levels of the genes was observed in the group treated with 0.70 mg/L of Cu^2+^ (*p* < 0.05; Figure 3A).

In the gills, the mRNA levels of *caspase-3*, *Bax*, *p53*, and *cytc1* increased in treatments with 0.04 and/or 0.18 mg/L of Cu^2+^ and then decreased to near normal values in treatment with 0.70 mg/L of Cu^2+^ (Figure 3B). The *caspase-3* and *cytc1* were markedly upregulated under exposure to 0.04 and 0.18 mg/L of Cu^2+^ (*p* < 0.05; Figure 3B) and gradually decreased under exposure to 0.70 mg/L of Cu^2+^. Similarly, *Bax* and *p53* were upregulated after exposure to 0.04 mg/L of copper (*p* < 0.05) and gradually decreased with increasing Cu^2+^ concentrations (Figure 3B).

In the muscle, *caspase-3* and *caspase-8* transcription were significantly upregulated compared to the control group after 5 days of Cu^2+^ exposure (*p* < 0.05; Figure 3C). However, the mRNA levels of *Bax*, *p53*, and *cytc1* were not significantly altered after copper exposure.

### 3.3. Alterations in the Expression of MAPK Pathway-Related Genes

**Figure 4:**
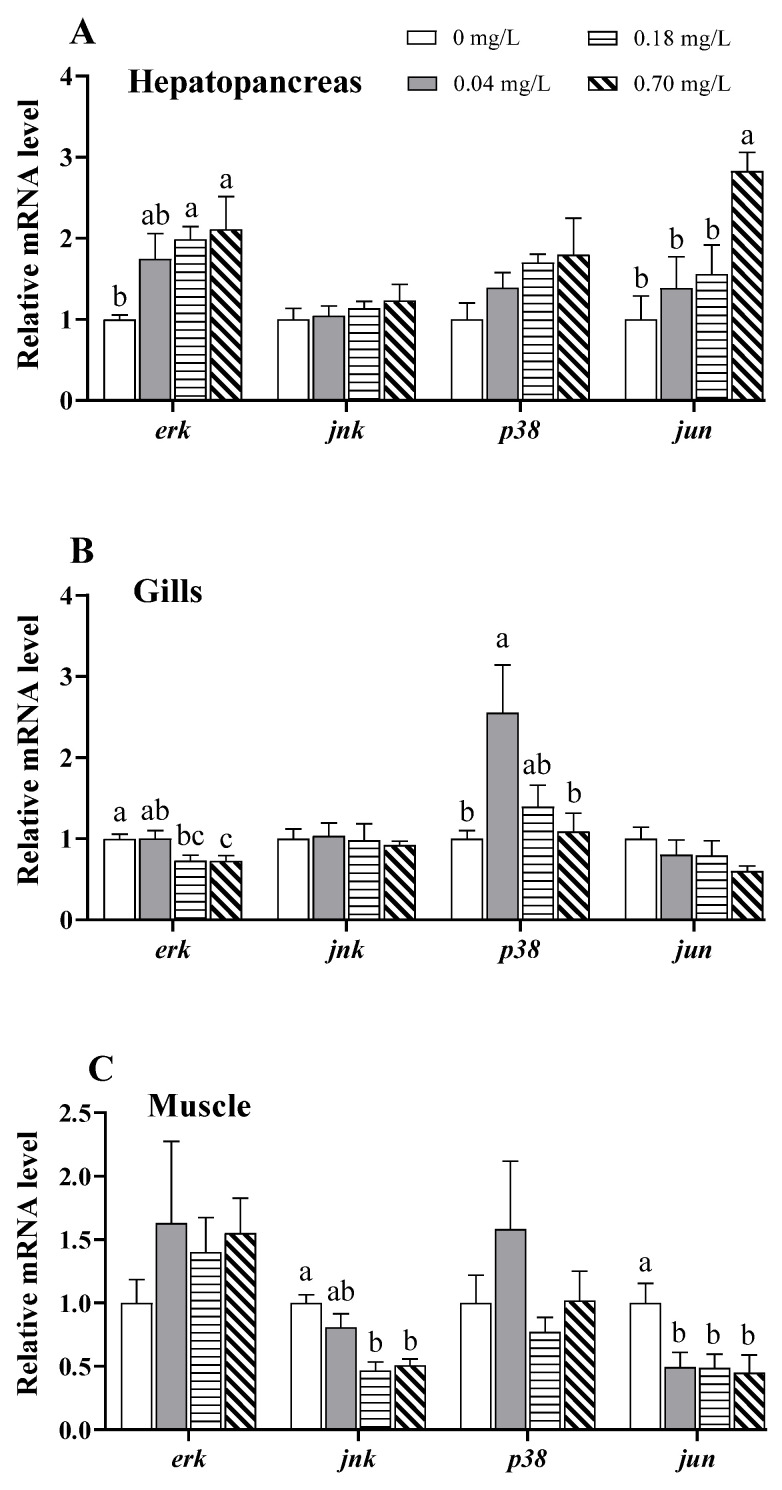
Expression of MAPK pathway-related genes in different tissues of *E. sinensis* exposed to copper for 5 days. (**A**) Hepatopancreas; (**B**) gills; (**C**) muscle. The values are expressed as the mean ± SEM (*n* = 6). Different letters denote significant differences among different groups (*p* < 0.05).

After Cu^2+^ exposure, the genes associated with the MAPK signaling pathway showed various degrees of change (Figure 4). In the hepatopancreas, the transcription of extracellular signal-regulated protein kinase (*erk*) was elevated in the groups exposed to 0.18 and 0.70 mg/L of Cu^2+^, and *jun* (an AP-1 subunit) was elevated in the group exposed to 0.70 mg/L of Cu^2+^, both compared to that in the control group (*p* < 0.05; Figure 4A). 

In the gills, *erk* expression was distinctly downregulated in the group exposed to 0.70 mg/L of Cu^2+^ compared to that in the control group (*p* < 0.05; Figure 4B), while *p38* expression was upregulated in the group exposed to 0.04 mg/L of Cu^2+^ and gradually downregulated under exposure to 0.18 and 0.70 mg/L of Cu^2+^ (*p* < 0.05; Figure 4B).

In the muscle, c-Jun N-terminal kinase (*jnk*) mRNA was downregulated in the groups exposed to 0.18 and 0.70 mg/L of Cu^2+^, and *jun* mRNA was downregulated in the groups exposed to 0.04, 0.18, and 0.70 mg/L of Cu^2+^, compared to those in the control group (*p* < 0.05; Figure 4C).

### 3.4. Alterations in the Expression of ER Stress-Related Genes

**Figure 5:**
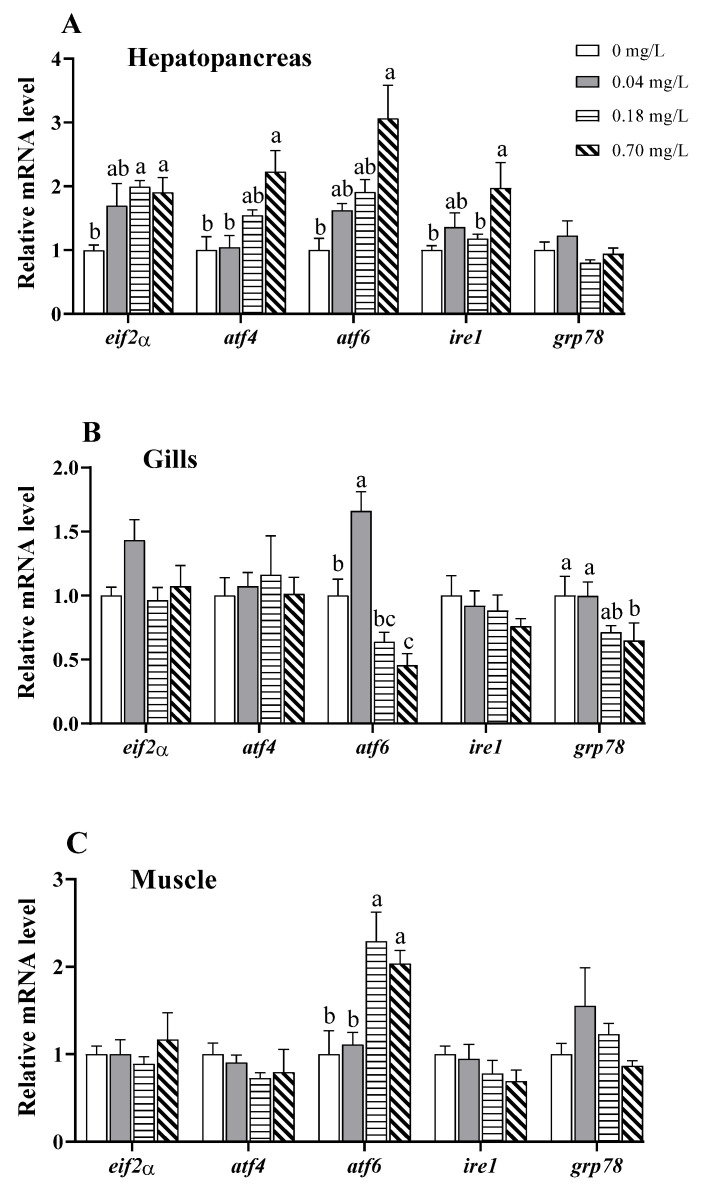
Expression of ER stress-related genes in different tissues of *E. sinensis* exposed to copper for 5 days. (**A**) Hepatopancreas; (**B**) gills; (**C**) muscle. The values are the mean ± SEM (*n* = 6). Different letters denote significant differences among different groups (*p* < 0.05).

The mRNA levels of the ER stress-related genes showed irregular variations after Cu^2+^ exposure in the hepatopancreas, gills, and muscle (Figure 5). In the hepatopancreas, the mRNA levels of activating transcription factor 6 (*atf6*) and *atf4* exhibited a linear rising trend with increasing Cu^2+^ concentrations and were upregulated in the group treated with 0.70 mg/L of Cu^2+^ (*p* < 0.05; Figure 5A). Compared to those in the control group, exposure to 0.18 and 0.70 mg/L of Cu^2+^ upregulated the transcription of eukaryotic translation initiation factor 2 α (*eif2α*), and 0.70 mg/L of Cu^2+^ upregulated inositol-requiring enzyme 1 (*ire1*) transcription (*p* < 0.05; Figure 5A). 

In the gills, the mRNA levels of *atf6* exhibited an initial upregulation followed by a decreasing tendency, and a peak value was observed in the crabs exposed to 0.04 mg/L of Cu^2+^ (*p* < 0.05; Figure 5B). The expression of *atf6* was downregulated under exposure to 0.70 mg/L of Cu^2+^ relative to that in the control group (*p* < 0.05; Figure 5B). In addition, exposure to 0.70 mg/L of Cu^2+^ decreased *grp78* transcription (*p* < 0.05; Figure 5B).

In the muscle, the mRNA level of *atf6* was significantly enhanced in the 0.18 and 0.70 mg/L Cu^2+^-exposed groups compared to that in the control group (*p* < 0.05), but other genes were not significantly changed (Figure 5B).

### 3.5. Alterations in the Expression of Autophagy-Related Genes

**Figure 6:**
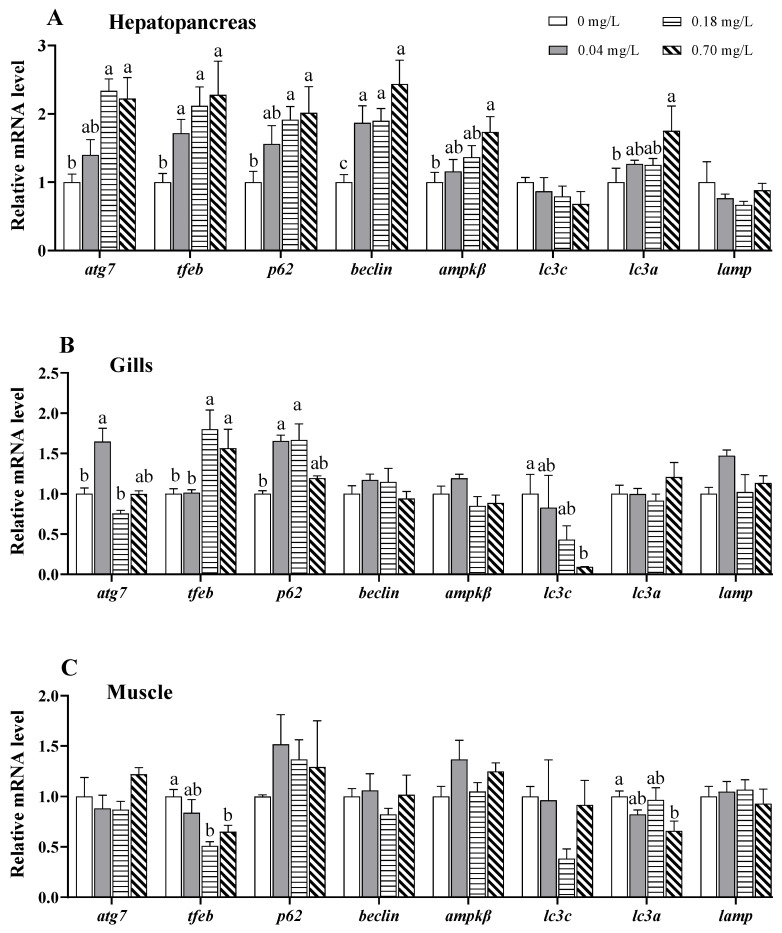
Expression of autophagy-related genes in different tissues of *E. sinensis* exposed to copper for 5 days. (**A**) Hepatopancreas; (**B**) gills; (**C**) muscle. The values are expressed as the mean ± SEM (*n* = 6). Different letters denote significant differences among different groups (*p* < 0.05).

Eight autophagy-related genes, including 5-AMP-activated protein kinase β (*ampkβ*), *beclin*, *p62*, microtubule-associated proteins 1A/1B light chain 3a (*lc3a*), *lc3c*, autophagy-related gene 7 (*atg7*), transcription factor EB (*tfeb*), and lysosome-associated membrane protein 1 (*lamp1*), were used to evaluate the autophagic response to Cu^2+^ exposure in the hepatopancreas, gills, and muscle (Figure 6). In the hepatopancreas, the mRNA levels of *atg7*, *tfeb*, *ampkβ*, *beclin*, *p62*, and *lc3a* increased with Cu^2+^ concentrations in a linear or non-linear manner, and they were significantly upregulated in the 0.70 mg/L Cu^2+^-exposed group compared to the control group (*p* < 0.05; Figure 6A). A significant upregulation was also observed in *atg7* under exposure to 0.18 mg/L of Cu^2+^ and in *tfeb* and *p62* under exposure to 0.04 and 0.18 mg/L Cu^2+^ (*p* < 0.05; Figure 6A).

In the gills, Cu^2+^ exposure caused a significant increase in the *atg7* mRNA level in the 0.04 mg/L Cu^2+^-exposed group, *tfeb* in the 0.18 and 0.70 mg/L Cu^2+^-exposed groups, and *p62* in the 0.04 and 0.18 mg/L Cu^2+^-exposed groups compared with those in the control group (*p* < 0.05; Figure 6B). In contrast, Cu^2+^ exposure caused a significant decrease in *lc3c* mRNA in the 0.70 mg/L Cu^2+^-exposed group (*p* < 0.05; Figure 6B). 

In the muscle, only the transcription of *tfeb* and *lc3a* was significantly changed by Cu^2+^ exposure (Figure 6C). The transcription of *tfeb* was lower in the 0.18 and 0.70 mg/L Cu^2+^-exposed groups than in the 0 mg/L Cu^2+^-exposed group (*p* < 0.05; Figure 6C). Furthermore, the transcription of *lc3a* was lower in the 0.70 mg/L Cu^2+^-exposed group than in the 0 mg/L Cu^2+^-exposed group (*p* < 0.05; Figure 6C).

### 3.6. Alterations in the Expression of Immune Response-Related Genes

**Figure 7:**
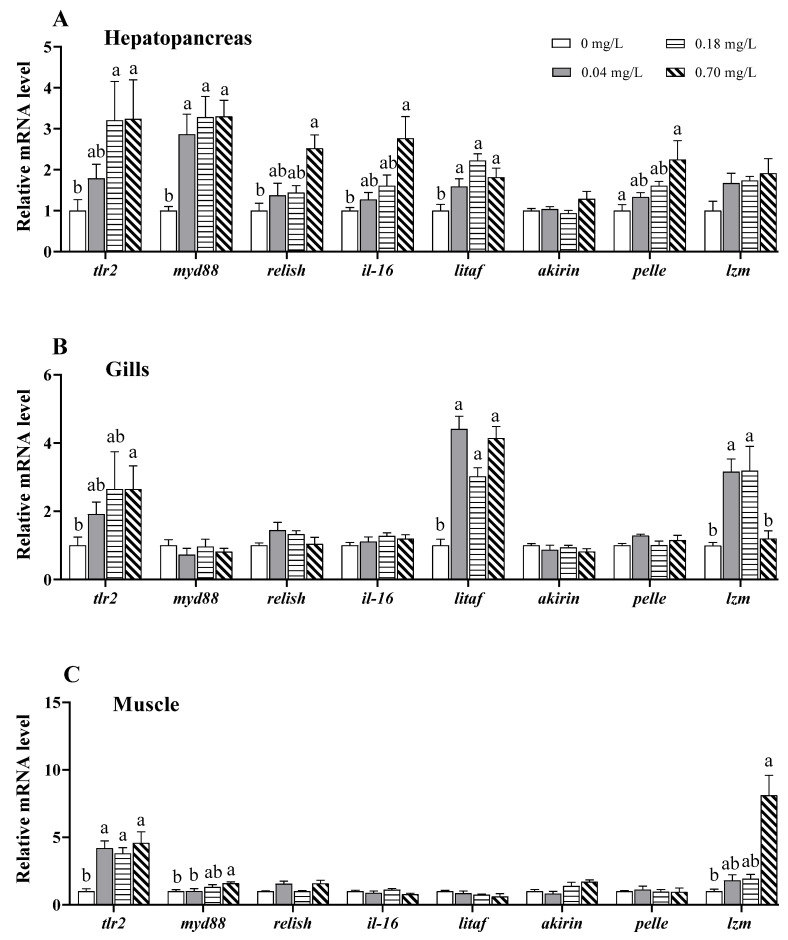
Expression of immune response-related genes in different tissues of *E. sinensis* exposed to copper for 5 days. (**A**) Hepatopancreas; (**B**) gills; (**C**) muscle. The values are expressed as the mean ± SEM (*n* = 6). Different letters denote significant differences among different groups (*p* < 0.05).

The immune response to Cu^2+^ exposure was assessed by determining the immune response-related genes in the hepatopancreas, gills, and muscle (Figure 7). In the hepatopancreas, the mRNA levels of Toll-like receptor 2 (*tlr2*), myeloid differentiation protein-88 (*myd88*), *relish*, interleukin-16 (*il-16*), lipopolysaccharide-induced TNF-α factor (*litaf*), and *pelle* were higher in the 0.70 mg/L Cu^2+^-exposed group than in the control group (*p* < 0.05; Figure 7A). Higher mRNA levels of *tlr2* and *myd88* were also observed in the 0.18 mg/L Cu^2+^-exposed group (*p* < 0.05; Figure 7A).

In the gills, the mRNA level of *tlr2* was strongly upregulated in the 0.70 mg/L Cu^2+^-treated group compared to that in the control group (*p* < 0.05; Figure 7B). The *litaf* in the three Cu^2+^-treated groups and lysozyme (*lzm*) in the 0.04 and 0.18 mg/L Cu^2+^-treated groups were highly expressed (*p* < 0.05; Figure 7B). 

In the muscle, the transcription of *tlr2* was upregulated in the three Cu^2+^-treated groups compared to that in the control group (*p* < 0.05; Figure 7C). Likewise, the expression of *myd88* and *lzm* was upregulated in the 0.70 mg/L Cu^2+^-treated group (*p* < 0.05; Figure 7C).

### 3.7. Alterations in the Expression of Stress- and Detoxification-Related Genes

**Figure 8:**
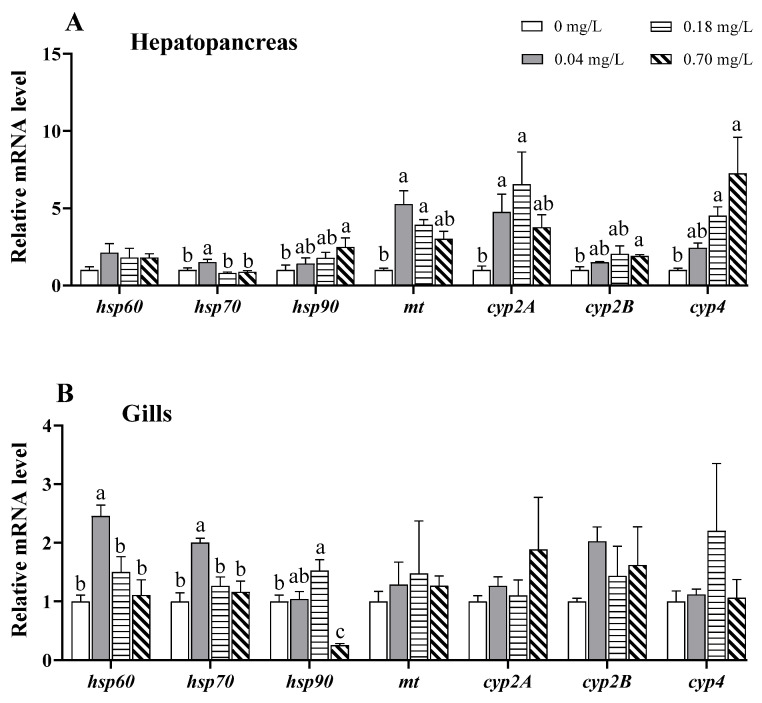

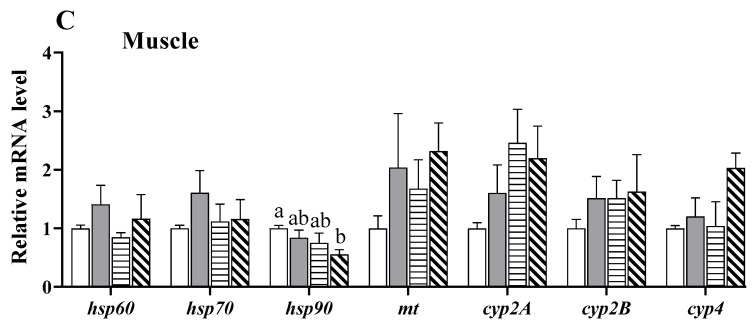
Expression of stress- and detoxification-related genes in different tissues of *E. sinensis* exposed to copper for 5 days. (**A**) Hepatopancreas; (**B**) gills; (**C**) muscle. The values are expressed as the mean ± SEM (*n* = 6). Different letters denote significant differences among different groups (*p* < 0.05).

In the hepatopancreas, the mRNA levels of shock protein 90 (*hsp90*), cytochrome P450 (*cyp*) 2b, and *cyp4* exhibited a linear rising trend with increasing Cu^2+^ concentrations, and upregulation was observed in the 0.70 mg/L Cu^2+^-treated group (*p* < 0.05; Figure 8A). The mRNA levels of *hsp70* and metallothioneins (*mt*) were first upregulated and then downregulated with increasing Cu^2+^ concentrations, as evidenced by higher *hsp70* expression under exposure to 0.04 mg/L of Cu^2+^ and higher *mt* and *cyp2a* expression under exposure to 0.04 and 0.18 mg/L of Cu^2+^ (*p* < 0.05; Figure 8A).

In the gills, the mRNA levels of *hsp60* and *hsp70* were significantly upregulated under 0.04 mg/L Cu^2+^ exposure (*p* < 0.05; Figure 8B) but gradually reduced to the same level as that in the control group. Similarly, *hsp90* expression was upregulated in the group exposed to 0.18 mg/L of Cu^2+^ but downregulated in the group exposed to 0.70 mg/L of Cu^2+^ (*p* < 0.05; Figure 8B). Other genes were not markedly affected by Cu^2+^ exposure.

In the muscle, only *hsp90* expression was significantly reduced in the 0.70 mg/L Cu^2+^-exposed group compared to that in the control group (*p* < 0.05; Figure 8C).

## 4. Discussion

Excess copper has been widely confirmed to be toxic to crustaceans, and the toxic effect is linked not only to concentration but also to exposure time. The median lethal concentration (24–96 h LC_50_) of Cu^2+^ decreased with the extension of exposure time in crustaceans [12,13]. Exposure to 0.75 mg/L Cu^2+^ for 7 days resulted in abnormal gill tip structure of *M. rosenbergii* [29]. The stress biomarkers showed an increased tendency in a time-dependent manner (1–7 days) in *Macrobrachium scabriculum* exposed to Cu^2+^ at doses of 0.032–0.352 mg/L [30]. A study of 3–48 h of exposure showed that Cu^2+^ treatments (5–20 mg/L) began to negatively influence the immune ability of *L. vannamei* after 12 h [31]. Similar to previous studies, our data also exhibited that exposure to Cu^2+^ (0.04–0.70 mg/L) for 5 days had adverse effects on antioxidative status, apoptosis, ER stress, and immune response in *E. sinensis*. It is worth noting that the Cu^2+^ toxicity showed tissue-specificity, and hepatopancreas was more sensitive to Cu^2+^ exposure in *E. sinensis.* In invertebrates, metals, including copper, are commonly taken in via gills and accumulate in the hepatopancreas [32]. Yang et al. reported that the accumulation of copper in the hepatopancreas was higher than in other tissues in *E. sinensis* after Cu^2+^ exposure [33]. Meanwhile, the hepatopancreas is considered a primary organ of excretion and detoxification for metals in crustaceans [34]. Thus, it may be more susceptible to copper exposure. 

### 4.1. Effects of Copper Exposure on Antioxidative Status

Oxidative stress is a physiological imbalance state in which the production of reactive oxygen species (ROS) overwhelms the cellular antioxidant defense capacity, eventually resulting in damage to cellular macromolecules, such as DNA, proteins, and lipids. Copper is known to participate in the formation of ROS, and its overload may result from repetitive radical formation via redox cycling [35,36]. Excessive ROS can induce oxidative stress and impair the antioxidant defense system. Indeed, strong evidence exists that acute or chronic Cu^2+^ exposure induces oxidative stress in different aquatic animals. For example, Cu^2+^ exposure enhances the activities of antioxidative enzymes such as SOD and glutathione peroxidase (Gpx) in hepatopancreas of *L. vannamei* [37] and *Callinectes sapidus* [38], and gills of *O. niloticus* [39], reflecting an occurrence of oxidative stress. In contrast, exposure to high levels of waterborne Cu^2+^ decreases enzymatic and non-enzymatic antioxidants and induces oxidative damage in the gills of *P. clarkia* [40], the hepatopancreas of *Minuca rapax* [41], and the brain of *Cyprinus carpio* [42]. Our study further showed that the antioxidant capacity in different tissues of *E. sinensis* decreased following exposure to 0.70 mg/L of Cu^2+^, indicating that a higher level of Cu^2+^ exposure induces oxidative damage. In addition, our data showed a variable intensity of oxidative stress in different tissues after Cu^2+^ exposure, which was supported by a previous study in *Carassius auratus* [43], indicating the tissue specificity of Cu^2+^ toxicity.

Peroxidative damage to membrane lipids is another common consequence of excess Cu^2+^. Lipid peroxy radicals formed during lipid peroxidation may change the fluidity and permeability of the cell membrane in injured cells [44]. MDA, a lipid peroxidation product, is a typical indicator used to evaluate lipid peroxidation. It is increased in multiple fish tissues after Cu^2+^ exposure [45,46,47]. Similarly, Cu^2+^-overloaded *Procambarus clarkii* has a significantly increased MDA concentration in the hemolymph, hepatopancreas, and gills [40,48,49]. Our data also exhibited enhanced MDA content in the hemolymph, gills, and muscle of *E. sinensis* after exposure to 0.70 mg/L of Cu^2+^, indicating that high levels of Cu^2+^ exposure induce lipid peroxidation and augment oxidative damage.

It has been reported that the toxic effect of copper on redox state was related to cultured conditions, such as salinity, temperature, and pH, in aquatic animals. Moderate salinity levels increased GST activity to alleviate the lethal toxicity of Cu^2+^, but high salinity levels worsen the Cu^2+^-induced oxidative damage in *Danio rerio* embryos [50]. In *M. rapax,* the higher temperature (35 °C) significantly increased Cu^2+^-induced oxidative stress [41]. Carvalho et al. (2015) suggested that the effect of Cu^2+^ on the response of antioxidant defense systems was determined by water pH in *Prochilodus lineatus* [51]. The evidence revealed that copper combined with other factors causes more significant toxicity in aquatic animals than copper alone. Thus, interactive effects between copper exposure and cultured conditions will be examined in future research. 

### 4.2. Effects of Copper Exposure on Apoptosis

Apoptosis is considered a sensitive parameter for assessing the toxicity of environmental pollutants [52]. It has been reported that Cu, a common environmental pollutant, can induce apoptosis in aquatic animals. High concentrations of Cu^2+^ increase the incidence of TUNEL-positive cells (apoptosis) in the gills of *D. rerio* and *C. auratus* [53,54]. Acute exposure to Cu^2+^ increases the apoptotic hemocyte ratio and *caspase-3* gene expression in *L. vannamei* [55]. In our study, apoptosis-related genes such as *caspase-3*, *caspase-8*, *Bax*, *p53*, and *cytc* were upregulated in the hepatopancreas, gills, and/or muscle of *E. sinensis*, indicating that mitochondria-mediated apoptosis was activated by Cu^2+^ exposure. Cu^2+^-induced apoptosis is likely elicited by the induction of ROS [56]. Our data support this view, given the strong oxidative stress that was found after Cu^2+^ exposure. Additionally, in *D. rerio*, the central nervous system and liver show higher sensitivity to apoptosis induced by Cu^2+^ exposure [57]. Similarly, Cu^2+^-exposed *E. sinensis* exhibits stronger apoptosis in the hepatopancreas and gills. In the hepatopancreas, the activation of apoptosis was mainly observed under exposure to 0.7 mg/L of Cu^2+^, while in the gills, it was mainly observed under exposure to 0.04 and 0.18 mg/L of Cu^2+^. Thus, Cu-triggered apoptosis may occur in a tissue-specific manner.

MAPK signaling pathways, including ERK, JNK, and p38, play critical roles in apoptosis [58]. The ERK-AP-1 and JNK-AP-1 pathways have been reported to regulate oxidative stress-induced apoptosis [59]. A previous study reported that Cu^2+^ exposure causes apoptosis via the activation of ERK and p38 in the hepatocytes of *O. mykiss* [60]. Mitochondrial apoptosis induced by copper nanoparticles has been associated with the activation of the ERK signaling pathway in female mice [61]. In our study, the mRNA levels of *erk* and *jun* (a AP-1 subunit) were upregulated in the hepatopancreas after Cu^2+^ exposure and significantly associated with apoptosis, indicating that the ERK-AP-1 pathway may be involved in Cu^2+^-induced apoptosis. In the gills, *p38* gene expression was upregulated in the 0.04 mg/L copper-exposed group, which implies that the p38 pathway may be activated to regulate apoptosis after exposure to lower dose of Cu^2+^. In the muscle, however, the mRNA levels of *jnk* and *jun* were downregulated after Cu^2+^ exposure, although the underlying mechanisms remain unclear. We hypothesize that the downregulation may be related to tissue damage caused by Cu^2+^ exposure.

### 4.3. Effects of Copper Exposure on ER Stress 

The ER is a pivotal organelle that is responsible for protein assembly, folding, and transportation. Protein misfolding and ER stress trigger a complex signaling process, known as the unfolded protein response (UPR), to restore ER homeostasis [62]. A triggered UPR is a protective mechanism to reinstate ER homeostasis, but persistent or severe ER stress can initiate cell death via mitochondrial pathways [63]. Environmental pollutants, such as Cu^2+^, activate ER stress and impair mitochondrial function in aquatic animals [64]. Cu^2+^ exposure for 30 days leads to upregulated ER stress-related genes, such as *grp78*, *perk*, *eif2a*, *ire-1α*, and *atf6* in the liver of *Synechogobius hasta* and *Pelteobagrus fulvidraco* [65,66]. We also observed a marked upregulation of *eif2a*, *atf4*, *atf6*, and *ire1* in the hepatopancreas of *E. sinensis*, indicating that exposure to 0.7 mg/L of Cu^2+^ induced ER stress. In the gills, exposure to 0.04 mg/L of Cu^2+^ upregulated *atf6*, while 0.7 mg/L of Cu^2+^ downregulated *atf6* and *grp78*. We hypothesize that the downregulation of these genes was related to ER damage under exposure to higher concentrations of Cu^2+^. Similar data have also been found in the liver of *S. hasta* exposed to a higher level (0.055 mg/L) of Cu^2+^ for 60 days [65]. In addition, increased ROS production under Cu^2+^ exposure can induce ER stress and activate the ATF6 and IRE1 signaling pathways, leading to apoptosis [67].

### 4.4. Effects of Copper Exposure on Autophagy 

Autophagy is a crucial cell-clearing process that regulates the degradation of damaged organelles and unfolded proteins by fusion with lysosomes in cells. LC3 and p62 are widely used as markers of autophagy. In the later stages of autophagy, TFEB coordinates lysosomal activation and autophagosome–lysosome fusion [68]. A recent study reported that excess dietary copper induces oxidative stress and autophagy, as evidenced by the upregulated expression of *beclin1*, *lc3B*, and *p62* in *P. fulvidraco*, which then protected against copper-induced lipid accumulation [69]. Activated autophagy has also been reported in GC-1 cells [70], pig testes [71], and the hypothalamus of broilers [72] following Cu^2+^ exposure due to oxidative stress. In contrast, Cu^2+^ exposure has been found to downregulate the mRNA levels of *lc3* in *D. rerio* gills, indicating the impairment of macroautophagy [73]. Furthermore, the AMPK signaling pathway has been shown to regulate Cu^2+^-induced autophagy [74,75]. In our study, the mRNA levels of autophagy-related genes, including *ampkβ*, *beclin*, *lc3a*, *tfeb*, *p62*, and *atg7*, were upregulated in the hepatopancreas, suggesting that Cu^2+^ exposure may activate autophagy via the AMPK-Beclin pathway. Unlike those in the hepatopancreas, the mRNA levels of *atg7* and *p62* in the gills were upregulated following exposure to lower doses of Cu^2+^ (0.04 and/or 0.18 mg/L) but returned to similar levels as those in the control group after being exposed to a higher dose of Cu^2+^ (0.7 mg/L). The expression of *lc3c* was even downregulated in the 0.7 mg/L Cu^2+^-exposed gills. The findings suggest that a low dose of Cu^2+^ may initiate autophagy, but a high dose can impair the autophagic process in the gills. The detailed mechanisms require further study. The activation of autophagy may be linked to oxidative stress and ER stress induced by Cu^2+^ exposure [76].

### 4.5. Effects of Copper Exposure on the Immune Response

The immune response is a key mechanism following pollutant toxicity in aquatic organisms. TLRs, widely existing pattern-recognition receptors, are considered major regulators of the immune response [77]. Numerous studies have suggested that environmental pollutants, including heavy metals, can activate the TLRs to regulate immune response in animals. For example, Cr(VI) exposure upregulated *tlr2* and *myd88* expression in *Geloina erosa* gills [78], and microbiota-dependent TLR2 signaling reduced silver nanoparticle toxicity to *D. rerio* larvae [79]. Relish, an NF-κB transcription factor, also plays a key role in the innate immunity of crustaceans [80]. In crustaceans, the TLR2-MyD88 pathway regulates the immune response to pathogenic bacterial infections [81,82]. A transcriptomic analysis revealed that Cu^2+^ exposure significantly affects the TLR pathway in *Mizuhopecten yessoensis* [83]. In *L. vannamei*, the gene expression of TLRs was significantly increased in the 0.05 mg/L Cu^2+^-treated group, but returned to the control level following treatments with higher doses of Cu^2+^ [37]. Aksakal and Ciltas [84] also reported that a low expression of immune-related genes such as *tlr4* and *tlr22* resulted in immunosuppression in *D. rerio* after exposure to copper oxide nanoparticles. In our study, the immune response to Cu^2+^ exposure was tissue-specific. In the hepatopancreas, a significant inflammatory response occurred via the TLR2-MyD88-NF-κB pathway after Cu^2+^ exposure. Despite the upregulation of *tlr2* and/or *myd88* in the gills and muscle, *relish* and *il-16* (an important pro-inflammatory cytokine in crabs) were not altered, which may indicate no obvious inflammatory response in the two tissues, especially the muscle. In addition, the ERK pathway may also be involved in Cu^2+^-induced inflammation in the hepatopancreas [85].

In addition to NF-κB, LITAF is a pivotal transcription factor in the inflammatory response and regulates the transcription of TNF-α and other cytokines [86]. Tang et al. [87] suggested that LITAF is a mediator from the NF-κB pathway in the lipopolysaccharide-induced inflammatory response. It has been reported that *litaf* is upregulated and involved in the immune response in *E. sinensis* after *Edwardsiella tarda* and *Vibrio anguillarum* infections [86]. Similarly, our data show that Cu^2+^ exposure upregulated the expression of *litaf* in the hepatopancreas, suggesting that the TLR2-MyD88-LITAF pathway may be triggered in response to Cu^2+^ toxicity.

### 4.6. Effects of Copper Exposure on the Stress Response and Detoxification

HSPs are molecular chaperones that play well-established roles in protein folding and transport. HSP60, HSP70, and HSP90 are well-studied HSPs that are abundantly induced under a variety of chemical exposures [88], which is a protective response to stressors [89]. A previous study reported that Cu^2+^ exposure upregulated the mRNA levels of *hsp60*, *hsp70*, and *hsp90* in the liver of *C. carpio* [90]. A study on freshwater prawns (*Macrobrachium malcolmsonii*) further showed that the synthesis of HSP70 appeared from the 1st to 24th hour in the gills under Cu^2+^ exposure but was not recorded after the 24-h mark [91]. We also observed that the mRNA levels of *hsp60*, *hsp70*, and *hsp90* exhibited an initial upregulation followed by a decreasing tendency in the gills. We therefore conjectured that Cu^2+^ exposure at lower doses triggered an HSP-mediated protective mechanism. In addition, the downregulation of *hsp90* may be interpreted as a result of the strong oxidative stress induced by higher doses of Cu^2+^ [92]. 

MT, a metal-binding protein with a high affinity for metals, is involved in the regulation of essential metal ion homeostasis and the detoxification of non-essential metal ions [93]. After 4 days of Cu^2+^ exposure, the MT level was found to increase in *Gasterosteus aculeatus* [94]. An increased MT level has also been reported after Cu^2+^ exposure in *Pacifastacus leniusculus* [95] and constitutes a protective response to Cu^2+^ accumulation. Similarly, our data show upregulated *mt* expression in the hepatopancreas after exposure to 0.04 and 0.18 mg/L of Cu^2+^, indicating that a lower concentration of Cu^2+^ induces a positive response, but a higher concentration inhibits the response. 

CYP enzymes have been implicated in the detoxification and metabolism of environmental pollutants, including heavy metals, in aquatic animals. The CYP 1–4 families are considered reliable biomarkers for monitoring environmental toxicants [96]. The induction of CYP enzymes may be an adaptive response to metal exposure, whereas their decrease inhibits detoxification [97]. In Cu^2+^-exposed *Diaphanosoma celebensis*, CYP-related genes, including *cyp2* and *cyp4*, were found to be upregulated at an earlier exposure time (6 h) but downregulated at a later exposure time (24h) [98]. In *C. auratus*, Cu^2+^ exposure upregulated the expression of *cyp1a* and *cyp3a*, but combined treatment with Cu^2+^ and diclofenac decreased their expression [99]. In our study, the mRNA levels of *cyp2A, cyp2B*, and *cyp4* were markedly increased in the hepatopancreas, implying that CYP enzymes may be involved in the phase I detoxification of Cu^2+^ toxicity. In addition, our data revealed that detoxification predominantly occurred in the hepatopancreas but not in the gills or muscle.

Apart from acute toxicity, long-term Cu^2+^ exposure also causes its accumulation in different tissues of crustaceans. In *E. sinensis*, the copper accumulation was positively related to its level in water, and the hepatopancreas was the primary target organ [33]. The accumulation presents potential for bio-magnification through the food chain [100], which may pose a health risk to humans, since humans are the primary consumers of *E. sinensis* and other aquatic animals. In order to maintain cellular homeostasis, many organisms possess a purification ability of toxic elements. Boada et al. [101] reported that *Mugil curema* eliminated enriched copper for14 days after Cu^2+^ exposure. Similarly, the purification process in juvenile *Petenia kraussii* exposed to Cu^2+^ was achieved after 14 days [102]. In *P. clarkii*, enrichment of copper in hepatopancreas was completely eliminated after 7 days [103]. However, the depuration time of *E. sinensis* for excessive copper has not been reported until now, and will be further evaluated in our future research. Furthermore, the potential health risks of copper accumulation from aquatic food consumption should be investigated.

## 5. Conclusions

In this study, we examined the adverse effects of Cu^2+^ exposure on different tissues of *E. sinensis*. Cu^2+^ exposure suppressed antioxidative parameters and promoted lipid peroxidation in different tissues, resulting in oxidative damage. After Cu^2+^ exposure, apoptosis-related genes were upregulated, implying that apoptosis was activated, and the activation may be related to the upregulation of the MAPK pathway and ER stress. In the hepatopancreas and gills, the regulation of autophagy-related genes indicated that the autophagic response was involved in Cu^2+^ toxicity. In addition, Cu^2+^ exposure increased immune-related gene expression in different tissues, especially the hepatopancreas, where the TLR2-MyD88-NF-κB pathway may be initiated to mediate the inflammatory response. Furthermore, the upregulation of anti-stress and detoxification genes revealed that an adaptive mechanism was activated in different tissues following Cu^2+^ exposure. Overall, the toxicity response of Cu^2+^ in *E. sinensis* was associated with oxidative stress, apoptosis, ER stress, autophagy, and immune response. This study enriches our understanding of the potential toxicity response of Cu^2+^ in crustaceans, which may provide more reference data for the environmental risk assessments of Cu^2+^.

## Data Availability

All data are included in the manuscript and Supplementary Materials.

## Supplementary Materials

The following supporting information can be downloaded at: https://www.mdpi.com/article/10.3390/antiox11102029/s1, Table S1: Specific primer sequences for qPCR in the study.

## References

[1] Tapiero H., Townsend D.á., Tew K. (2003). Trace elements in human physiology and pathology. Copper. Biomed. Pharmacother..

[2] Ugarte M., Osborne N.N., Brown L.A., Bishop P.N. (2013). Iron, zinc, and copper in retinal physiology and disease. Surv. Ophthalmol..

[3] Rehman M., Liu L.J., Wang Q., Saleem M.H., Bashir S., Ullah S., Peng D.X. (2019). Copper environmental toxicology, recent advances, and future outlook: A review. Environ. Sci. Pollut. Res..

[4] O’Gara B.A., Bohannon V.K., Teague M.W., Smeaton M.B. (2004). Copper-induced changes in locomotor behaviors and neuronal physiology of the freshwater oligochaete, Lumbriculus variegatus. Aquat. Toxicol..

[5] Tavares-Dias M. (2021). Toxic, physiological, histomorphological, growth performance and antiparasitic effects of copper sulphate in fish aquaculture. Aquaculture.

[6] Zhang B.H., Ding Z.G., Li H.Q., Mou X.Z., Zhang Y.Q., Yang J.Y., Zhou E.M., Li W.J. (2016). Algicidal Activity of Streptomyces eurocidicus JXJ-0089 Metabolites and Their Effects on Microcystis Physiology. Appl. Environ. Microbiol..

[7] Chen W.Y., Ju Y.R., Lin C.J., Tsai J.W., Chen S.C., Liao C.M. (2015). Environmental stochasticity promotes copper bioaccumulation and bioenergetic response in tilapia. Stoch. Environ. Res. Risk Assess..

[8] Zhang F., Li D., Yang Y., Zhang H., Zhu J., Liu J., Bu X., Li E., Qin J., Yu N. (2022). Combined effects of polystyrene microplastics and copper on antioxidant capacity, immune response and intestinal microbiota of Nile tilapia (*Oreochromis niloticus*). Sci. Total Environ..

[9] Daglish R.W., Nowak B.F. (2002). Rainbow Trout Gills Are a Sensitive Biomarker of Short-Term Exposure to Waterborne Copper. Arch. Environ. Contam. Toxicol..

[10] Brown V., Dalton R. (1970). The acute lethal toxicity to rainbow trout of mixtures of copper, phenol, zinc and nickel. J. Fish Biol..

[11] Mohamed M., El-Fiky S., Soheir Y., Abeer A. (2008). Cytogenetic studies on the effect of copper sulfate and lead acetate. pollution on Oreochromis niloticus fish. J. Cell Biol..

[12] Dai X., Zang W., Yang H., Zhong X., Jiang M., Ke X. (2001). The toxic effects of Cu^2+^,Zn^2+^,Cd^2+^ on giant freshwater prawn juvenile. J. Shanghai Fish. Univ..

[13] Dong X., Lv L., Wang A., Wang L., Miao J. (2010). Study on the acute toxicity of Cu^2+^ and Cd^2+^ acting on Procambarus clarkii juvenile. J. Hydroecology.

[14] Yao Q., Zang W.l., Dai X., Jiang M., Xu G., Ding F. (2003). A acute toxic effects of copper, cadmium, dichlorvos and methamidophos on Penaeus vannamei larval shrimp and their interactions. J. Shanghai Fish. Univ..

[15] Tilton F.A., Bammler T.K., Gallagher E.P. (2011). Swimming impairment and acetylcholinesterase inhibition in zebrafish exposed to copper or chlorpyrifos separately, or as mixtures. Comp. Biochem. Physiol. Part C Toxicol. Pharmacol..

[16] Van Aardt W., Hough M. (2006). Acute effects of Cu on oxygen consumption and 96 hr-LC50 values in the freshwater fish *Tilapia sparrmani* (Teleostei: Cichlidae) in Mooi River hard water, South Africa. Afr. J. Aquat. Sci..

[17] Rougier F., Troutaud D., Ndoye A., Deschaux P. (1994). Non-specific immune response of Zebrafish, Brachydanio rerio (Hamilton-Buchanan) following copper and zinc exposure. Fish Shellfish Immunol..

[18] Hoseini S.M., Hoseinifar S.H., Doan H.V. (2018). Effect of dietary eucalyptol on stress markers, enzyme activities and immune indicators in serum and haematological characteristics of common carp (*Cyprinus carpio*) exposed to toxic concentration of ambient copper. Aquac. Res..

[19] Vani Paila R., Rao Yallapragada P., Thatipaka S.D.R. (2012). Response of glutathione system and carotenoids to sublethal copper in the postlarvae of Penaeus indicus. Ecotoxicol. Environ. Saf..

[20] Frías-Espericueta M.G., Castro-Longoria R., Barrón-Gallardo G.J., Osuna-López J.I., Abad-Rosales S.M., Páez-Osuna F., Voltolina D. (2008). Histological changes and survival of Litopenaeus vannamei juveniles with different copper concentrations. Aquaculture.

[21] Tang D., Liu R., Shi X., Shen C., Bai Y., Tang B., Wang Z. (2021). Toxic effects of metal copper stress on immunity, metabolism and pathologic changes in Chinese mitten crab (*Eriocheir japonica sinensis*). Ecotoxicology.

[22] Sun S., Ge X., Hu J., Yu H., Jiang Z. (2014). Effects of water-borne copper on the survival, antioxidant status, metallothionein-I mRNA expression and physiological responses of the *Chinese mitten crab*, *Eriocheir sinensis* (Decapoda: Brachyura) larvae. Sci. Mar..

[23] Yang Z.B., Zhao Y.L., Zhou Z., Yang J. (2006). Effects of water CuSO4 concentration on molting, growth and survival of eriocheir sinensis. Acta Hydrobiol. Sin..

[24] Zhou C. (2021). Toxic Effects of Copper Ions on Eriocheir sinensis.

[25] Jia R., Du J., Cao L., Feng W., He Q., Xu P., Yin G. (2020). Chronic exposure of hydrogen peroxide alters redox state, apoptosis and endoplasmic reticulum stress in common carp (*Cyprinus carpio*). Aquat. Toxicol..

[26] Livak K., Schmittgen T. (2001). Analysis of relative gene expression data using real-time quantitative PCR and the 2(-Delta Delta C(T)) Method. Methods–A Companion Methods Enzymol..

[27] Huang S. (2017). Study on Reciprocal Regulation Mechanism of MIH and Ec R Genes in the Molting Process of Chinese Mitten Crab, Eriocheir Sinensis.

[28] Sanchez W., Burgeot T., Porcher J.-M. (2013). A novel “Integrated Biomarker Response” calculation based on reference deviation concept. Environ. Sci. Pollut. Res..

[29] Asih A.Y.P., Irawan B., Soegianto A. (2013). Effect of copper on survival, osmoregulation, and gill structures of freshwater prawn (Macrobrachium rosenbergii, de Man) at different development stages. Mar. Freshw. Behav. Physiol..

[30] Mukherjee A. (2022). Experimental study of copper toxicity and some stress biomarkers in *Macrobrachium scabriculum* (Heller, 1862). J. Appl. Aquac..

[31] Yeh S.T., Liu C.H., Chen J.C. (2004). Effect of copper sulfate on the immune response and susceptibility to *Vibrio alghiolyticus* in the white shrimp *Litopenaeus vannamei*. Fish Shellfish Immunol..

[32] Grosell M., Blanchard J., Brix K., Gerdes R. (2007). Physiology is pivotal for interactions between salinity and acute copper toxicity to fish and invertebrates. Aquat. Toxicol..

[33] Yang Z., Zhao Y., Zhou Z., Zhou X., Yang J. (2005). Effects of copper in water on distribution of copper and digestive enzymes activities in *Eriocheir sinensis*. J. Fish. CHINA.

[34] Gurkan M. (2018). Effects of three different nanoparticles on bioaccumulation, oxidative stress, osmoregulatory, and immune responses of *Carcinus aestuarii*. Toxicol. Environ. Chem..

[35] Bremner I. (1998). Manifestations of copper excess. Am. J. Clin. Nutr..

[36] Gaetke L.M., Chow C.K. (2003). Copper toxicity, oxidative stress, and antioxidant nutrients. Toxicology.

[37] Qian D., Xu C., Chen C., Qin J.G., Chen L., Li E. (2020). Toxic effect of chronic waterborne copper exposure on growth, immunity, anti-oxidative capacity and gut microbiota of Pacific white shrimp *Litopenaeus vannamei*. Fish Shellfish Immunol..

[38] Brouwer M., Brouwer T.H. (1998). Biochemical defense mechanisms against copper-induced oxidative damage in the blue crab, *Callinectes sapidus*. Arch. Biochem. Biophys..

[39] Ma’rifah F., Saputri M.R., Soegianto A., Irawan B., Putranto T.W.C. (2019). The Change of Metallothionein and Oxidative Response in Gills of the *Oreochromis niloticus* after Exposure to Copper. Animals.

[40] Wei K., Yang J. (2015). Oxidative damage induced by copper and beta-cypermethrin in gill of the freshwater crayfish *Procambarus clarkii*. Ecotoxicol. Environ. Saf..

[41] Capparelli M.V., Bordon I.C., Araujo G., Gusso-Choueri P.K., de Souza Abessa D.M., McNamara J.C. (2019). Combined effects of temperature and copper on oxygen consumption and antioxidant responses in the mudflat fiddler crab *Minuca rapax* (Brachyura, Ocypodidae). Comp. Biochem. Physiol. Part C Toxicol. Pharmacol..

[42] Jiang W.-D., Liu Y., Hu K., Jiang J., Li S.-H., Feng L., Zhou X.-Q. (2014). Copper exposure induces oxidative injury, disturbs the antioxidant system and changes the Nrf2/ARE (CuZnSOD) signaling in the fish brain: Protective effects of myo-inositol. Aquat. Toxicol..

[43] Husak V.V., Mosiichuk N.M., Kubrak O.I., Matviishyn T.M., Storey J.M., Storey K.B., Lushchak V.I. (2018). Acute exposure to copper induces variable intensity of oxidative stress in goldfish tissues. Fish Physiol. Biochem..

[44] Qian Y., Buettner G.R. (1998). The EPR detection of lipid-dervied radicals during membrane lipid peroxidation of cells. Free Radic. Biol. Med..

[45] Rajabiesterabadi H., Hoseini S.M., Fazelan Z., Hoseinifar S.H., Hien Van D. (2020). Effects of dietary turmeric administration on stress, immune, antioxidant and inflammatory responses of common carp (*Cyprinus carpio*) during copper exposure. Aquac. Nutr..

[46] Kong X., Jiang H., Wang S., Wu X., Fei W., Li L., Nie G., Li X. (2013). Effects of copper exposure on the hatching status and antioxidant defense at different developmental stages of embryos and larvae of goldfish *Carassius auratus*. Chemosphere.

[47] Kamunde C., MacPhail R. (2011). Effect of humic acid during concurrent chronic waterborne exposure of rainbow trout (*Oncorhynchus mykiss*) to copper, cadmium and zinc. Ecotoxicol. Environ. Saf..

[48] Wei K., Yang J. (2016). Copper-induced oxidative damage to the prophenoloxidase-activating system in the freshwater crayfish *Procambarus clarkii*. Fish Shellfish Immunol..

[49] Wei K., Yang J. (2015). Oxidative damage of hepatopancreas induced by pollution depresses humoral immunity response in the freshwater crayfish *Procambarus clarkii*. Fish Shellfish Immunol..

[50] Santos B., Andrade T., Domingues I., Ribeiro R., Soares A.M.V.M., Lopes I. (2021). Influence of salinity on the toxicity of copper and cadmium to *Zebrafish embryos*. Aquat. Toxicol..

[51] Carvalho C.d.S., Bernusso V.A., Fernandes M.N. (2015). Copper levels and changes in pH induce oxidative stress in the tissue of curimbata (*Prochilodus lineatus*). Aquat. Toxicol..

[52] AnvariFar H., Amirkolaie A.K., Jalali A.M., Miandare H.K., Sayed A.H., Üçüncü S.İ., Ouraji H., Ceci M., Romano N. (2018). Environmental pollution and toxic substances: Cellular apoptosis as a key parameter in a sensible model like fish. Aquat. Toxicol..

[53] Vergolyas M.R., Veyalkina N.N., Goncharuk V.V. (2010). Effect of copper ions on hematological and cytogenetic parameters of freshwater fishes *Carassius auratus gibelio*. Cytol. Genet..

[54] Luzio A., Monteiro S.M., Fontaínhas-Fernandes A., Pinto-Carnide O., Matos M., Coimbra A.M. (2013). Copper induced upregulation of apoptosis related genes in zebrafish (*Danio rerio*) gill. Aquat. Toxicol..

[55] Guo H., Li K.X., Wang W., Wang C.G., Shen Y.C. (2017). Effects of Copper on Hemocyte Apoptosis, ROS Production, and Gene Expression in White Shrimp *Litopenaeus vannamei*. Biol. Trace Elem. Res..

[56] Chandra J., Samali A., Orrenius S. (2000). Triggering and modulation of apoptosis by oxidative stress. Free Radic. Biol. Med..

[57] Hernandez P.P., Undurraga C., Gallardo V.E., Mackenzie N., Allende M.L., Reyes A.E. (2011). Sublethal concentrations of waterborne copper induce cellular stress and cell death in zebrafish embryos and larvae. Biol. Res..

[58] Junttila M.R., Li S.-P., Westermarck J. (2008). Phosphatase-mediated crosstalk between MAPK signaling pathways in the regulation of cell survival. FASEB J..

[59] Hiramatsu N., Kasai A., Yao J., Meng Y., Takeda M., Maeda S., Kitamura M. (2004). AP-1-independent sensitization to oxidative stress-induced apoptosis by proteasome inhibitors. Biochem. Biophys. Res. Commun..

[60] Nawaz M., Manzl C., Lacher V., Krumschnabel G. (2006). Copper-induced stimulation of extracellular signal-regulated kinase in trout hepatocytes: The role of reactive oxygen species, Ca(^2+^), and cell energetics and the impact of extracellular signal-regulated kinase signaling on apoptosis and necrosis. Toxicol. Sci..

[61] Zhang C.H., Wang Y., Sun Q.Q., Xia L.L., Hu J.D., Cheng K., Wang X., Fu X.X., Gu H. (2018). Copper Nanoparticles Show Obvious in vitro and in vivo Reproductive Toxicity via ERK Mediated Signaling Pathway in Female Mice. Int. J. Biol. Sci..

[62] Schröder M., Kaufman R.J. (2005). ER stress and the unfolded protein response. Mutat. Res. Fundam. Mol. Mech. Mutagen..

[63] Kim I., Xu W., Reed J.C. (2008). Cell death and endoplasmic reticulum stress: Disease relevance and therapeutic opportunities. Nat. Rev. Drug Discov..

[64] Xu Y.-H., Xu Y.-C., Hogstrand C., Zhao T., Wu L.-X., Zhuo M.-Q., Luo Z. (2020). Waterborne copper exposure up-regulated lipid deposition through the methylation of GRP78 and PGC1α of grass carp *Ctenopharyngodon idella*. Ecotoxicol. Environ. Saf..

[65] Song Y.-F., Huang C., Shi X., Pan Y.-X., Liu X., Luo Z. (2016). Endoplasmic reticulum stress and dysregulation of calcium homeostasis mediate Cu-induced alteration in hepatic lipid metabolism of javelin goby Synechogobius hasta. Aquat. Toxicol..

[66] Song Y.-F., Luo Z., Zhang L.-H., Hogstrand C., Pan Y.-X. (2016). Endoplasmic reticulum stress and disturbed calcium homeostasis are involved in copper-induced alteration in hepatic lipid metabolism in yellow catfish *Pelteobagrus fulvidraco*. Chemosphere.

[67] Zhao G., Sun H., Zhang T., Liu J.-X. (2020). Copper induce zebrafish retinal developmental defects via triggering stresses and apoptosis. Cell Commun. Signal..

[68] Settembre C., Di Malta C., Polito V.A., Arencibia M.G., Vetrini F., Erdin S., Erdin S.U., Huynh T., Medina D., Colella P. (2011). TFEB Links Autophagy to Lysosomal Biogenesis. Science.

[69] Zhong C.-C., Zhao T., Hogstrand C., Chen F., Song C.-C., Luo Z. (2022). Copper (Cu) induced changes of lipid metabolism through oxidative stress-mediated autophagy and Nrf2/PPARγ pathways. J. Nutr. Biochem..

[70] Kang Z.L., Qiao N., Liu G.Y., Chen H.M., Tang Z.X., Li Y. (2019). Copper-induced apoptosis and autophagy through oxidative stress-mediated mitochondrial dysfunction in male germ cells. Toxicol. Vitr..

[71] Li Y.L., Chen H.M., Liao J.Z., Chen K.L., Javed M.T., Qiao N., Zeng Q.W., Liu B.X., Yi J.N., Tang Z.X. (2021). Long-term copper exposure promotes apoptosis and autophagy by inducing oxidative stress in pig testis. Environ. Sci. Pollut. Res..

[72] Liao J.Z., Yang F., Chen H.L., Yu W.L., Han Q.Y., Li Y., Hu L.M., Guo J.Y., Pan J.Q., Liang Z.P. (2019). Effects of copper on oxidative stress and autophagy in hypothalamus of broilers. Ecotoxicol. Environ. Saf..

[73] Luzio A., Parra S., Costa B., Santos D., Álvaro A., Monteiro S. (2021). Copper impair autophagy on zebrafish (*Danio rerio*) gill epithelium. Environ. Toxicol. Pharmacol..

[74] Liao J.Z., Yang F., Yu W.L., Qiao N., Zhang H., Han Q.Y., Hu L.M., Li Y., Guo J.Y., Pan J.Q. (2020). Copper induces energy metabolic dysfunction and AMPK-mTOR pathway-mediated autophagy in kidney of broiler chickens. Ecotoxicol. Environ. Saf..

[75] Chen H.L., Wang Y.Y., Luo J., Kang M., Hou J., Tang R.P., Zhao L., Shi F., Ye G., He X.L. (2022). Autophagy and apoptosis mediated nano-copper-induced testicular damage. Ecotoxicol. Environ. Saf..

[76] Fornai F., Puglisi-Allegra S. (2021). Autophagy status as a gateway for stress-induced catecholamine interplay in neurodegeneration. Neurosci. Biobehav. Rev..

[77] Fan Z.-J., Zou P.-F., Yao C.-L. (2015). Toll-like receptors (tlr) and its signaling pathway in teleost. Acta Hydrobiol. Sin..

[78] Wang G., Zhang C., Huang B. (2020). Transcriptome analysis and histopathological observations of *Geloina erosa* gills upon Cr(VI) exposure. Comp. Biochem. Physiol. Part C Toxicol. Pharmacol..

[79] Brinkmann B.W., Koch B.E.V., Peijnenburg W.J.G.M., Vijver M.G. (2022). Microbiota-dependent TLR2 signaling reduces silver nanoparticle toxicity to zebrafish larvae. Ecotoxicol. Environ. Saf..

[80] Zhu F., Sun B.Z., Wang Z.Y. (2019). The crab Relish plays an important role in white spot syndrome virus and *Vibrio alginolyticus* infection. Fish Shellfish Immunol..

[81] Gao Q., Tang Q., Xia Z., Yi S., Cai M., Du H., Yang J., Li J., Xing Q., Luo J. (2021). Molecular identification and functional analysis of MyD88 in giant freshwater prawn (*Macrobrachium rosenbergii*) and expression changes in response to bacterial challenge. Int. J. Biol. Macromol..

[82] Jiang Z., Li X., Gao X., Jiang Q., Chen Q., Zhang S., Tong S., Liu X., Zhu J., Zhang X. (2020). Pathogenicity of Aeromonas hydrophila causing mass mortalities of *Procambarus clarkia* and its induced host immune response. Microb. Pathog..

[83] Meng X.L., Tian X., Nie G.X., Wang J.L., Liu M., Jiang K.Y., Wang B.J., Guo Q.Q., Huang J.R., Wang L. (2015). The transcriptomic response to copper exposure in the digestive gland of Japanese scallops (*Mizuhopecten yessoensis*). Fish Shellfish Immunol..

[84] Aksakal F.I., Ciltas A. (2019). Impact of copper oxide nanoparticles (CuO NPs) exposure on embryo development and expression of genes related to the innate immune system of zebrafish (*Danio rerio*). Comp. Biochem. Physiol. Part C Toxicol. Pharmacol..

[85] Park J.-Y., Chung T.-W., Jeong Y.-J., Kwak C.-H., Ha S.-H., Kwon K.-M., Abekura F., Cho S.-H., Lee Y.-C., Ha K.-T. (2017). Ascofuranone inhibits lipopolysaccharide–induced inflammatory response via NF-kappaB and AP-1, p-ERK, TNF-α, IL-6 and IL-1β in RAW 264.7 macrophages. PLoS ONE.

[86] Li S., Jia Z., Li X., Geng X., Sun J. (2014). Identification and expression analysis of lipopolysaccharide-induced TNF-alpha factor gene in Chinese mitten crab *Eriocheir sinensis*. Fish Shellfish Immunol..

[87] Tang X.R., Metzger D., Leeman S., Amar S. (2006). LPS-induced TNF-alpha factor (LITAF)-deficient mice express reduced LPS-induced cytokine: Evidence for LITAF-dependent LPS signaling pathways. Proc. Natl. Acad. Sci. USA.

[88] Gupta S.C., Sharma A., Mishra M., Mishra R.K., Chowdhuri D.K. (2010). Heat shock proteins in toxicology: How close and how far?. Life Sci..

[89] Singh M.P., Reddy M.M., Mathur N., Saxena D.K., Chowdhuri D.K. (2009). Induction of hsp70, hsp60, hsp83 and hsp26 and oxidative stress markers in benzene, toluene and xylene exposed *Drosophila melanogaster*: Role of ROS generation. Toxicol. Appl. Pharmacol..

[90] Jiang X.Y., Guan X.T., Yao L.L., Zhang H., Jin X., Han Y. (2016). Effects of Single and Joint Subacute Exposure of Copper and Cadmium on Heat Shock Proteins in Common Carp (*Cyprinus carpio*). Biol. Trace Elem. Res..

[91] Yamuna A., Kabila V., Geraldine P. (2000). Expression of heat shock protein 70 in freshwater prawn *Macrobrachium malcolmsonii* (H. Milne Edwards) following exposure to Hg and Cu. Indian J. Exp. Biol..

[92] Jia R., Du J., Cao L., Feng W., He Q., Xu P., Yin G. (2021). Immune, inflammatory, autophagic and DNA damage responses to long-term H_2_O_2_ exposure in different tissues of common carp (*Cyprinus carpio*). Sci. Total Environ..

[93] Amiard J.C., Amiard-Triquet C., Barka S., Pellerin J., Rainbow P.S. (2006). Metallothioneins in aquatic invertebrates: Their role in metal detoxification and their use as biomarkers. Aquat. Toxicol..

[94] Santos E.M., Ball J.S., Williams T.D., Wu H., Ortega F., Van Aerle R., Katsiadaki I., Falciani F., Viant M.R., Chipman J.K. (2010). Identifying health impacts of exposure to copper using transcriptomics and metabolomics in a fish model. Environ. Sci. Technol..

[95] Gunderson M.P., Boyd H.M., Kelly C.I., Lete I.R., McLaughlin Q.R. (2021). Modulation of endogenous antioxidants by zinc and copper in signal crayfish (*Pacifastacus leniusculus*). Chemosphere.

[96] Uno T., Ishizuka M., Itakura T. (2012). Cytochrome P450 (CYP) in fish. Environ. Toxicol. Pharmacol..

[97] Aziz N., Butt A. (2020). Enzymatic and non-enzymatic detoxification in Lycosa terrestris and *Pardosa birmanica* exposed to single and binary mixture of copper and lead. Environ. Toxicol. Pharmacol..

[98] Han J., Lee K.-W. (2021). Identification and response of cytochrome P450 genes in the brackish water flea *Diaphanosoma celebensis* after exposure to benzo [α] pyrene and heavy metals. Mol. Biol. Rep..

[99] Xie Z., Luan H., Zhang Y., Wang M., Cao D., Yang J., Tang J., Fan S., Wu X., Hua R. (2020). Interactive effects of diclofenac and copper on bioconcentration and multiple biomarkers in crucian carp (*Carassius auratus*). Chemosphere.

[100] Naqvi S., Devalraju I., Naqvi N. (1998). Copper bioaccumulation and depuration by red swamp crayfish, *Procambarus clarkii*. Bull. Environ. Contam. Toxicol..

[101] Barron M.G., Adelman I.R. (1984). Nucleic acid, protein content, and growth of larval fish sublethally exposed to various toxicants. Can. J. Fish. Aquat. Sci..

[102] Lemus M., Chung K. (1999). Effect of Temperature on Copper Toxicity, Accumulation, and Purification in Tropical Fish Juveniles *Petenia kraussii* (Pisces: Cichlidae). Caribb. J. Sci..

[103] Dan Z., Zhang X., Liu D., Ru S. (2019). Cu accumulation, detoxification and tolerance in the red swamp crayfish *Procambarus clarkii*. Ecotoxicol. Environ. Saf..

