# Supplementary material for: Effects of Copper Exposure on Oxidative Stress, Apoptosis, Endoplasmic Reticulum Stress, Autophagy and Immune Response in Different Tissues of Chinese Mitten Crab (Eriocheir sinensis)
Source: Antioxidants (Basel). 2022 Oct 14;11(10):2029. doi: 10.3390/antiox11102029 (PMC9598082; doi:10.3390/antiox11102029)
Supplement: Supplementary file 1 [file antioxidants-11-02029-s001.zip › antioxidants-1896804-supplementary.pdf]

Table S1. Specific primer sequences for qPCR in the study

Type	Gene	Primer sequence (5'-3')	Genbank number
apoptosis-related genes	*caspase-3*	F: GAGCCACGGCACGAACACC	MH183147.1
		R: AGGCTGGGGCACTGCTCATC	
	caspase-8	F: TGGAGCGTCATGGTTCAGAC	AKS36884.1
		R: CAGACAAGCCACCACTGCTA	
	bax	F: TGCTGTTGCCTCCACTTACC	Sequence No.1 from our RNA-seq
		R: ATGAGGGGTCTCGACTGGAA	
	bcl-2	F: CATCATCTCCCTCTTCGCGG	Sequence No.2 from our RNA-seq
		R: CAGTCCCATCACGTCGATCA	
	p53	F: TCGACATGGAAGGGAAGCAC	JQ613218.1
		R: CTGACTTCAAACGGCACAGC	
	cytc1	F: GGCCAAGGATGTGTCAGTGT	Sequence No.3 from our RNA-seq
		R: AGTACGGACCACTTGTGTCG	
MAPK pathway-related		F: CGCGAGTTGCAGATCCAGAA	
genes	*erk*		GU002542.1
		R: CAAGGGGCGATTGGACAACA	
	jnk	F: ACCTCCTGTCTCGCATGTTG	KC900087
		R: ACAGTGTGTTCCCGGTCATC	
	p38	F: TACGGGCAAGTCTGCTCGGC	KF582665.1
		R: AAGGTGGTGGAGGGCGTGAAG	
	AP-1 (jun)	F: CCCGCACCTACAAACTCCAT	Sequence No.4 from our RNA-seq
		R: CTGCAATGCGGTTCCTTAGC	
endoplasmic reticulum			
stress-related genes	*eif2α*	F: GCTGACAGTGTAACCACCCT	Sequence No.5 from our RNA-seq
		R: TGTGGAGTCCCCTTGGATGT	
	atf4	F: ATTCCAACCTTGTCCCTGCC	Sequence No.6 from our RNA-seq
		R: GTCTTCAGGGTAGACGTGCC	
	atf6	F: CAACCTTGGACCCTTGTCGG	Sequence No.7 from our RNA-seq
		R: CTGCGTCATCCCTTGACCAC	
	ire1	F: TAGTTTTGTGGTCCTGCCCC	Sequence No.8 from our RNA-seq
		R: GGACTTGGTGATGGCATGGA	
	grp78	F: CCTTCTATGCCACCAAGCGA	KC493626.1
		R: CTTGCCATCGTCGGTCTGTA	
autophagy-related genes	*atg7*	F: TCCGACTTCATCCGAAAATACC	MT543027.1
		R: GCACTCAACCCCAAGCCTG	
	tfeb	F: CCAACAACAGTGTGCCTGAC	Sequence No.9 from our RNA-seq
		R: TGAACCTTCGTCTTCGCTCA	
	p62	F: ACAGACGCCAAGTACCAAGG	Sequence No.10 from our RNA-seq
		R: AGGCTCACTCGTCTCCTGAT	
	beclin	F: GCCCATATACTGTGGCGAGG	MH173046.1

		R: CCAGGTCAAAGAGCCCAGTT	
	ampkβ	F: CAATCGTTGACCTCCCAGAA	MK676045.1
		R: ACTTCCCTTTCCTTCCCAGAG	
	lc3c	F: CACGTTGCCTATCCTCGACA	Sequence No.11 from our RNA-seq
		R: GTCATCGTCCCTACACTCGC	
	lc3a	F: ACGTCACGATGGGAGAACTG	Sequence No.12 from our RNA-seq
		R: GTGGTGGTGCTCGTAAACCT	
	lamp	F: TACACTGCACTGCCGACAAC	Sequence No.13 from our RNA-seq
		R: TATGCTCCCGACCTTTTGCG	
immune response-related genes	*toll2*	F: GCATACCAGGACGACGAACA	KC011816.1
		R: TGAGCGAGGAGAGCTTGTTG	
	myd88	F: ACAAGAGTCAAACCGGGGAC	KM433864.1
		R: AGAACTGGTGGTGCTCATGG	
	relish	F: TCAGGATTCGGTGGCAACTC	GQ871279.1
		R: ATCTGCACTTGGACCGATGG	
	il-16	F: TGACATTGGTCGCTCGTCTC	AWM96383.1
		R: CTTGAGATGCGAGGGAGGTC	
	litaf	F: ATCAGCTCCCCCACCCTATG	KF892539.1
		R: GTTGTTGGAGCAGCACCTTG	
	akirin	F: CAAACCGCTCTTCACCTTCAG	KC011818.1
		R: AAGGGCTGTTTGGGAGAGTC	
	pelle	F: CCCTACACCGAGAGGAAGCA	KP795393.1
		R: GACTCGCCACACTTCTCAGC	
	lzm	F: ATGATGCGTGTGATCTGCC	JN416111.1
		R: CATGACGCATCCATCGCTTG	
stress and detoxification-related genes	*hsp60*		KP642083.1
		F: AAAGGCAAGGGCAAGTCGTC	
		R: GGCCATTCGTTCCTGCATCT	
	hsp70	F: GGCAAGGCAGCGAAGGTCATC	KC493625.1
		R: CGGCATTGGTGACAGACTGACG	
	hsp90	F: TCACCAACGACTGGGAGGAT	EU809924
		R: CAGGAAGAGGAGTGCCCTGA	
	mt	F: ACCTCCTGCCGCTGTACTCCCT	GU479376.1
		R: GGAAGACGGCGTTGGGAAGG	
	cyp2A	F: ACCGCCGCTTCACCTTAC	KR262517
		R: CTTGCTTGCCACCATCTGC	
	cyp2B	F: ACTTGGTCCTCCTCATCTTCG	KT159166
		R: TCTCCCTCAGCATCCTCTCC	
	cyp4	F: AAGACTTCGTGGAGGTGTTC	KX812713
		R: GCACAGCGTTATGTTGGTGAAG	

Internal reference	*β-actin*	F: GCATCCACGAGACCACTTACA	HM053699.1
		R: CTCCTGCTTGCTGATCCACATC	
	ube	F: TTGCGTTCACAACTCGTATCTACC	HQ436509
		R: GTCCGTGAGGAGGGAACAGA	

Gene sequences applied in this study

Sequence No.1 *bax*

ATGGCCAATGGGTCTATTGAATCAAAGGAAGAGGACAGTTTGTGGCCAGCAGATGACT
CAGCACAGTCGTTGCCCTCTTCCCGAAATCGTCAGGACTCTGGCACTTTAGGTGACCC
CAGTGACAACTTTTCACAGGTTGGAAGACACCTTCCCCCACTCTTACACCCACGTCCA
CCCTCACCAGTCTCTGTGGCAGAGCTGCAGGAACGTGCCGTTCAGGATAGCCAAAAC
AATAATGTTGATGAGAACAGCAATGACCACCATTTTGACCAGTATGCTGCTGCTGCTGT
TGCCTCCACTTACCCTCGTCGGGAAGCTTCATCTTACCCTGCATACCCTAATCAAGAGG
CTTCGCATTACTCATCCTCCTATAGAGATGCATCGTCATACCCTCCATACAACAGCAGAG
AAACTTCACATCCCAGCAGGGAAAGTTCTTCAAGCTTAAGTTGGGAAGCCTCATCACA
TGATGCCTGGAGAGCCTCACACCCCTCATCTTCCAGTCGAGACCCCTCATCTTTGCACT
CATACAGGGAAGCTTCACACCAAGAAGCTCAGTCACACCACCATTCACGTTCTTCACA
TTCCACTCAGTATACCTTACACTCAAGCTACCCTTCACACTCTGTTCATGAGGCTGTCTC
TGCCTCTGATCAGTCTGTGCGACCCAGGGTGAGGCAACCATCACAGCATCAGAGAGAT
GAAGCAGACCACGCCATACCTCCCCGGTTACGTCTTGCCCCAAGAAGGGACAGCTTTA
GCAGCAGCAGTTGCCAGCACAGTGGACTCACCCACCGTAGAAACCGTTCTGAAGGCA
CGTCACCTGTCAACAGGTACCCACCACTGCCAAATCATAACTGCCCTCCTAAAGAATC
AGATGGACGACTACCCCAACTACCAGGGTGTAGGACAGGGAACCAAAACCCCAACAG
CCCTATGAGATGGCTTCCCCTTGCCTTGCGTGGGGGCAGATGCAGCCCCCAGCCCCCTC
AGGACTTGCCTCCTGCTGAGAATGTTACTCAGGAGGCTCAGGAATTGCTCACCACCTT
CACCTTAGAGACCCTTCGTAACAACAGGCTGGATGTGCCTCAGTGTATGCAGGCCATG
CCCAGTTCCAGCAGCCCCCACATGATGCGTGCTGGCCGGGAACTACGTCTGCTGGCAG
ATGCCTTTGCAGGGACAGCTGAGCGACGGAAGGTGCGTCTTATGGCAGAGTCAGTGA
ACCTCAGCTGCATTGAGATGAGTGACTTCTTCCAGCTGTGTGCTGAACTGTTTTATGAC
GGTATCACACGAGAACGTATTGTGGCATTGTTCACCTTCGTGGGTGATGTGGCTGTGCA
CCAAGTAAGGATGAAGGGAGAGGAACTGATCCATCTGCTAATGAAGTGGTCCTTTAGG
TACCTGGTGGAGCACGTGTGTCGCTGGGTGCAGGAGGCAGGTGGCTGGGTAAGTGTC
ACATACAAGGCTAACTACCACATCACAAATCTCTCCACATAA

Sequence No.2 *bcl2* (partial)

ATGGCCGGTCTATCGCTGGAGGGTCTCGGACAACCGGGTGTGGGCGTGAACGGCAGC
ATGGGCGTGAGGATGAGGAAGTCTTCGGTGCCCATAATCTCGCCTCGGTCCCTCGAAA
TCCCTGCCGGCCACATGCCCCCAAAGTCAATCTCCAGAAGCGGTTCCCCGGCCAGACG
CCGGTTCTCCAACGTGAGCGACGCGGTGACCAGGAAAATTTCTACCACGATAGGCTGG
CGGAGCGTGAACGTGGAGGCGGTGGTGACACAGGCCAAATGCCTCCTGACGCAGTAC
ATCAGGTGTCGCCTCAAACGGGCCGGCCTCCTTCACAAGAAACTGGGTCTTCAGCGGC
TCCGATCCGTGGCCAACCTCGCGGGCGGCTGGGAAGTGTGCGAGGTGTTCCCACGCG

TGTCAGGCATCGGTCAGGAGCTGGAGAGGACCTACCCAAAGCTGTACAGTTCGGTGG
CGCGTCAGCTGTCCGTCTCCCTCACGTCAGATAAGGTGGTTCGCTCCGTCATCGTGCAG
GTGGCAAGTCAGGTGTTCCACGGGGAGGTGAACTGGGCCCGCATCATCTCCCTCTTCG
CGGTGGCGGCGGGTCTGGCCTCGGACTGTGTTAAGCAAGGGCACCCCGAGTACGTGG
CCGGGGTGATCGACGTGATGGGACTGGTGACGGAGAGGCACACGGCGCCCTGGATAG
CCTCGCAGGGCGGCTGGAGTTCGCTGCTGTCGTGGTCGCAAACGCCGGAGACGGAAG
GGTCAGCGCTACAAGTATTGGTGTTGGTCGTGGCGGGGCTCATCGTGGCGGCCTTCCT
CCTCATCCTGGCCATCAAATTCCTCGGGGAGCTGGTGACG

Sequence No.3 *cytc1* (partial)

ATTGCCAAGATGGCGGCGGTGGCCTGTAGGGGTGTTCGTGCCGTGCTTTTGAAGGCCC
CCGGCACGACGATAACAGCACCGACCGCCAAGTTTTCAACCCTGAGGCAAATGGCGG
CCACCAGGAAGGCTGTACTGGCAGCTCTGGGCGTCCTGGGCGGGGGTGCAGCGGGTG
TGGCCTACGCCCTCAACGAGTCAGTGAAGGCGTCTGACCTTACTCTCCACACCACCAC
CTTCCCCTGGGCCCACAATGGCATCTTCTCCTCCCTGGACCATGCCTCCATCCGCCGTG
GTTATGAGGTGTATAAGAACGTGTGCGCTGCCTGTCATTCCATGAAGTACATCGCCTTC
CGTAACCTGATCGGCGTCTCCCACACTGAGGCCGAGGCTAAGGAGGAGGCGATGGGT
GTGCAGATCACCGATGGGCCAGATGATGAAGGCAACATGTTCCAAAGGCCCGGCAAG
CTCTCTGACTACTTCCCGAGCCCCTACACCAATGATGAAGCTGCCCGCTCTGCTAATAA
TGGTGCACTGCCGCCTGACCTCTCTTACATCACTTCTGCTCGCCACGGAGGGGAGAAC
TACATCTTCTCACTGCTGACCGGCTACTGCGACCCCCCTGCCGGTGTTTCCGTGAGGGA
GGGACTCTACTACAACCCCTATTTCCCGGGTGGTGCTATTGGCATGGCCCAGGCACTCT
ACAATGAGGTCATGGAGTACAGCGACGGCACGCCAGCCACCGCCTCACAGATGGCCA
AGGATGTGTCAGTGTTCCTCAAGTGGGCCTCTGAGCCGGAGCACGACGACCGCAAGA
GGATGGCCCTGAAGGTGCTCATGATGTTCACGATGCTATTTACTGTCTCCTACTACATCA
AGCGACACAAGTGGTCCGTACTCAAGAGCAGGAAGATCGCATACAAGCCTCCTCAATA
G

Sequence No.4 *AP-1 (jun)*

ATGTACGAGGACGGCTATAACCAGTACTCGCGGGAGGGCCTCACGCAGATTAAGCGGC
GTCTGACCCTTGACCTGACCAGCCGCCCCTCCAAACGACAGCGGCAGGGCGGGGGCA
TGGCGGGGAACTTCAACCCACTCCTGACCTCACCTGACCTCAACCAACTGAAGCTGG
CGTCCCCGGAACTGGAGAGACTGATTATGCAACAAAGCGGAGCGGTTGTTCTCAGCG
GTAGCGGTGGTGGTGGTGGTGGTGGTGGTGTTACGGGCGGTGGTGTTACGGGCGGTG
GTGTTACGACCTCAGCCAACACCAACACCACCACCGTCACCACCAGCACCTCAAGCTC
ACAGTTCTTCTTCCCCAAAACCGCTACCGTCGAGGAGGAGGAATTCGCTAGGGGCTTT
GAAGACACTTTGGAACAGCTGCACCACCAAGAGGCATTAGCGGCGGCAGTGACCAGC
GGTGGATCAGGGCTTCAATACACGACCCTTGAAGTGCCAACCAGTCTCCCCGCACCTA
CAAACTCCATGGGGCTACTTTCACAGGGGCACATCAAGGAGGAACCCCAAACAGTGC
CTAGCGTGGGTGCCTCCCCGCCGGTGTCACCAATCAACATGGAGTGCCAGGAGAGAAT
TAAGCTTGAACGTAAGAGGCTAAGGAACCGCATTGCAGCTTCGAAGTGCCGCAGGAG
GAAGCTTGAACGGATAGGCCGGCTGGAGGATAAAGTTCGCATGCTCAAAGGCGAGAA
CGTGGAGCTGCAGAATGTTGTTAATAGACTTCGCGACCAGGTGTGCAGTTTGAAGCAG
GAAGTGATGGAACATGTCAACTCAGGCTGCCAGATTCCCTTTGTCACGACCCACCAGC

AGTAG

Sequence No.5 *eif2α*

ATGAGGGCTGTGGCGTGTCACACAAATGGAGGGATAATATTCTCAGATGGCAAGGCAG
AAGACAATGAAGACTTGCAGGGAAGAGTAAAGACCTTCATTTTTCCCAAGATGGGAG
TAAAATTGCATGGATTAAAGGTGGCCAAGTGCATTTGGCTGAATCACCAGATTGGACA
AAGATTGGATCATTGAAGCATCCTCGAGCACAAGAAATATGCTTCTCTTCCCAGGGAA
ATTACTTGGCTGTTTGGGAATCGTATGTCAAAACAACAGACCAGAGTCAAGTCCAACC
AAACTTGAGTGTATATGATGTGTGTGGGAAGAAGTTGCTCAAAGCTTTCTTTCAACAA
AGTCAAATAAACTGGGAGCCACAATGGACACATGATGAAACTATTTTTGCTCGGAATG
TCACCAATGAAGTGCATTTTTATAAAGCTGATGACCTTCAAAACATTGCTGTAAAGAAG
ATTCTCCCAAAAGTGCGATCCTTCAGTTTATCGCCATCCACTCAAAACCATCATGTCAC
CTTTTTTGTGCCAGGTGCAAAAGGGAGTCCAGCTTACGTTCATCTCTACCGTTATCCAA
ATTTCCAAGACACTACCAGTGCCTTGGCCAACAAGAGTTTTTTAAGGCTGACAGTGT
AACCACCCTCTGGAACAAGCAGTCCACGGCTTGTCTGCTTCTGACAGCTGCTGAGGTT
GACAAATCAGGCACCTCATACTATGGGGAGCAGATGCTGTTTTACCTGAACATCCAAG
GGGACTCCACAAGGATAACTTTTACTGCAAGCACCGTCCCATATATACCCCAACATGAA
AGCCCAGCAGCACCATGGGCCAAAGCAAAATCAGGAAATATTCATTCTGTTTGTTGGA
GCCCAAATGGACAAGAGTTCTTTGCTGTCTATGGAACGATGCCGTCCAAAGCAACAAT
GTTCAACCAGAAGTGTGAAGTTGTGGCAGAGTTTGGTGAGGGAGCCAGAAATACCGT
CAGTGTGAATCCAGTAGGAAACATTGTACTGGTGGGGGGATTTGGGAACATCTCGCCC
AGGTATGAGATGTGGGATATTAAATTGAACAAAAAGATTGGTGAAACTGAATGTCCTG
ACACTACACACCATAGCTGGTCTCCATGTGGCCAATACCTTCTGACGTCCACCTGTTAT
GCTCGTCTCAAGGTCAATAATGGATACAAGGTGTGGCATTACACTGCTGTCTTGCAGCA
AGAGACTTCTCATGAAGAACTTTATGAAGCTTCGTGGGTTCCATGCCCAGATGCAGCTC
AACCCTTTGCTATTTCCACCAAACCTGTTTCTGGAATCTCCTCTTCAACAGCAGTTGCT
TCAAAACAGAGGTATATCCCTCCTTCCCAAAGAGGAGGAAAAGGCATTGTTGGGGCCA
CAAATACAGAGAAGAAGTTTTTGAGTCAAATGGACAACCCACCAAAAGAAGAAGCAC
CCCAGCAGCTGTCTAAAGCAGCCCTGAAAAACAAAAAAAAGAGAGAAGCAGCAAAG
AAAAAACGGGAGGAGGAGGGAAAAGATGAAACCAGCAAACAGAGCACGGGATCAGC
CTCCACAGAACCAGGCATTCAGTCATGCGGCTCAAATGTTGAATTGACTGGAAATCCT
GAAAAAGATAAGAAGATAAGAAACATTAAGAAGAAACTTAATGCAATTGCAAAGCTTA
AAGGAGAGCTTGAGAGTGGGAAGACTTTAGAGAAAGATCAACTGGAAAAGATTGCCA
CAGAGGAAAAGCTGATGAAAGACCTGGAGGACCTGGTCATTTGA

Sequence No.6 *atf4*

ATGGAGGGGCGAGAGCTCCTTGAGCTGCCCGATGTAGGGCTGTTTGACAACATGCCAG
CCTCACAGCCCTCCACACCTCTGGAAGACAGCCTCATGAAGATGGATGAGGAGGAGG
AGGACTACTGCTTCACCATCAAGCCAGAAGACATGCTTGTACCTCTGTCTGAGCTGCA
GCCTCCCACCACACCCACTCCTCCTCCCCTGGCCTCCACTCCCCCTGCCAGCCCAGTAC
ACTGTTATTCCAACCTTGTCCCTGCCAACCCTCCCCAAGTCCATGCCAGTCCACCTCGA
ATTCCTGCTCCTGCCCCTGCTGCCCAAGTCCAAGCCAGCGCTCCCCTGCCCAAGATCAT
CATCTGCCGCAAGGTTGGCACGTCTACCCTGAAGACCAAGCCAGTCAAGGTGCACTAC
CAAGTCATGCCAGTCAAGAAAATGCCACAAGTCACACCTGGCACTCCCTTGGTGAGCC

AGACACCCCTGCCAGTCAAACAAGTCCCACAAATTTCCTGTGGCACTCCCTCCTCCAC
CCACACACCCCTGCCAGCCCCCACAGCTGCCCCAGGGAACAGCCTAGGAGGTCAAAC
GGTTGTCAGGTCCCTTGTAGCAAGTCAGGGCAAGCTTCCAACCACTGGTTCATACATG
GATGTGCCTCAGGTTACCGTGAGTGCCGAGGAGCTTCCACCATGCGATGCCGAGGAGC
TGATCAACGAGATGGTACAGATAATTGGCGACAACAACAATGCCAACACCTTCTTCCA
GGGCCTTGAGGTGGAGGAAGGCATGGAGGAGCTTCTTCTCAAACTGGAAGCCTCAAA
CAAAGCCAACTCTGAGCCTGACTCCCAAGCCTCCCTGTCCGTTCCTGTCCCATCTGCG
TCTCCCAGCAGCTTCGGCTCTGCCTCTCCTCATCCCTCGGAAACCACCTTCTCTGGCTC
CATATCCTCGCCGGCCCACTCTGAATCCACCTTCCCATCTTCCCCTCTCTCCCTCCCCTC
CCCCGTCCCTTCTTTGGGCTCCCTCTCCCCAGCACAGTCCACCAGCGACTCTCTCTTCC
CTCCCGATGAGACGAGCTCCACCGAGGACTGTCTGGAGGATTGGAGCCCCCTGGAGG
AGACACACTCATACTCTAAATCAACACGCAAGTCAAAATCCTCCCGCAGGACCTCATC
TGCCAGGAAAGCACCGTACCCAGAGGACCGCAAGGAGCGCAAGAAGGAGCAGAACA
AGCAGGCGGCGCTGCGATACAGGCAGAAGAAGAAGCAGGAGGAAGATGACCTGTTG
GGCAAGATTGAGGTGGAGGAGGAGCGTCAGGAGAAGCTCAGGGCCACCTACAAGGG
TCTGAAGCAAGAATTGGGCTACCTGAAGAAGATCATGCGGGAGATGTTCATGGCCAAG
GGAATGCTCTCAGAGGAGGCACTGAAGAAGGCTGCCTTGGCCAAGAAGACGGCTACG
AAATAG

Sequence No.7 *atf6*
ATGTCCCCGGCCTCCTCCTCCTCTTCATCCTCGTCTTGTAACTCAAATTCCTTCAAGTAT
GATGGTGATATGACAGCGGAGGAGATGCTGACGCGCACACTCTACCCTAACCTGGTGG
CGAGCCCCGGGCCTGAGCTGCGGGGGATGGAACCGGAGAGTCTGGTGAAGGAGGAG
CCGGCCTCTCCGCAACACATCGTGCAGCTGCCGCCCTCCCCCGACGACACCAAAGAC
ATCTGGGACCTGCTGTGTGGGGGCGACGTCAAGCCCAACGTCAAGGTGCTGGACACT
CCCCCAATCACCCCACCCCAGAGTGAGGCCTCCCCCCCACACAGCCCCCAGCCCCCTT
CCCCTGTGGCCATGGTGTTGGGTCAGCACCATCACCCCCTGCAGCAGCACCAACAGAA
GCCCTTGGTCAGCAGTAGTAGTGGATCTGGCAACCATCTCTCTCAGCCCATCAAAGTC
GTCACCATCACAACGCGGAATGGAGGGTCAGGACCTGGGCCAACAAAGGGTGGGCGT
GTCACCAAAACCATGAAAATTCAGCCCAAGGTGATGAGCCCGTCTTCTGTGCAGACAC
CCCAGCCACAGGTCATCAGTATTGTCAACTCCTCCTCTGTGCCCAAGCTGACCCTGCCC
AAGACTGCTGTGACGAGAACCCTCCCAGCAGCCAGCCCCAGAGTAGTGCAGGTGAAG
ACCCCCGTCAGCCAGCCCCCCGCCAGCACCTCCCCCATCACCAGCCCGCTCCCCTCCA
TCATCACTGCAGTCAGCAGTCCAGTATCAGTGACGACAACTGCACCGCCTTCAGCCCC
CCTGGCCCCCCTGGCGCCAGCTCTCACCCCGGGCATGAAGCAGGGCAGCATACCCGAT
CTCAAGGCATTCAAGAGACAGCAGAGGATGATCAAGAACCGGGAGTCTGCCAGCCTC
TCCCGGAAGAAGAAGAAGGAGTACCTCACTTCCCTAGAGGCCAGCATCTCCGACCTAC
AGAAAGAGAACCAGAAACTGAAGGAGGAAAATCTTCTGCTGCAGCAGCGCTTGTCAA
GTCTGGAGACGGAATGTGATTCTCTGCGTCGATCAGCTGGCCGAGCCTCAAACCGAAA
GACCACTACGGCTCTGTTTGCTCTCATCTTCCTGTTCTCTCTCAACCTTGGACCCTTGTC
GGGAATCCTCTTGGGTGGAGAGTCAAAGCTGGACTCTCTCAAGGGGATCCTGAGAGG
GAGTGATGAGCTGAAAATCCCTCCCAGCTTCTCGCAGCGGTCTCTCCTGTGGTCAAGG
GATGACGCAGCCGAGGATGGTGGAGCTTCGTCACAGCTAGGCACCAACACCTCGCAC
ACCTGCCCCATGTACATCAATGCCACGGAGAGTCTGAGGCTGGAAACTGAGCTCAGGG

ACTGGTTTGAACACAAGTTCCGACCTTCAAAGACAGACCGCAAGAAAGACGACATCA
CGGCCGCTGCTGCTCCTGCTTCCCCAAAGACCAAGGAAGCGCGGAAGGGGCTGGCAG
GGCACACAGGGGAGGCAGTCAAGGGGGTAACATCAGGAAGAACCAGAGAAGCTATC
AGTGAGGTGTGGAATGCCCTGGCCCCTCCCCACCCACCCCCAGCACACCTGTACCACT
ACATCCACCCTGAGGCCCTGCACGTTGACCCTCCTGCCAGGCCCGAGGAGCAGCACG
CTGTCCTAGCCTCGGCACCACGCCTGTCGTCCTTCCTTGAGGCAATCCAGCGCCGTGAT
GACACTTACTACGTGGTCTCCTTCTCCCCCGACCACCTCCTTGTCCCTGCCACAGCCCG
TAATGACTCGGCACGCCCCCGCATGTCCCTCCTCATGCCAACTCCACGGCCAATCAACG
AGTCCCTGGCTCCTCCCAGGGACCACGTGGCTCTCATGCAGATTGACTGTGAGGTGAT
GCACACCCGCCTTGTACACGTGTCTGAGGAGTTCATTCCTCCACACCTGCGTGCTGGT
GGCAGCAACGCCTCACATCCAACATCACAAGCTCCTCCCGAGACCCCCCACCCTGCTC
GTGGGGATTCCAGGCGACGGTCAAAGTCAAGACCATCACTGCCCACCCAGTAG

Sequence No.8 *ire1*
ATGAAGACTGGCGCGAGGCCCTTGATGGCGGTGGTAGCGGGGTTAGTTTTGTGGTCCT
GCCCCCCAACCACCGCCACGAGACACAGGGACGCTAATATTAAGGACAAGATCACGTC
AACTGAACTACTAGCAGAGAGCGAGGAAGGCACTCTGCTGGTGTCCACTCTTGATGGC
GTGCTCCATGCCATCACCAAGTCCACGGGAGCCATCAAGTGGACCCTCAATGACGATC
CAATAATCATCAACCCAACAGATTCTTCACAACCGCCAATACCCCAGTTCTTCCCAAAT
CCCCAAGATGGTTCCCTCTACCGCTACACCATGAGTCGGGGACGTGACCCTCTCAAGA
AGCTGCCTTTCACCATTCCTCAGCTTGTGGCAAACTCTCCCTGTCGCTCCAGTGATGGC
ATATTCTACTTAGGTAAGAAGGTTGACTCTTGGGTTGGCATTGACCCCGTGACTGGAGA
GAAGCAGCTGGTCCTTGGTGTGGATGGTGTGAACCGCAATTGTCCCAAGCCTTCTCGC
AACACGGTTTACTTTGGCCGCACTCTGTACAACATCATACTATATGAGGCAGCAACTCA
GAACAAGTGGAACATCAGCTTCTCTGAATATGCTACCAGTTCCTCATCCACTATAGAAA
ACTATGACTTGGTGCACTACACTTCTACGGGTGAAGGCTCCATCCTCACCGTGGATCAG
CGCACCGGCTCCCTGCTGTGGACCCTGGAGCTGCCCTCCCCCCTGGTGGCCATGTACC
TGCTGGGGCCTGATGGTGGACTGCTCAGTGTTCCCTTGACCACAGTGGCTCCACAGAC
ACTGGAACACTTCACTCGGGAGTTCATGCACCTCACTCACCACAGCTCGGATGGCTCA
GAGGGCCTGCTCATCAAAAAGTTAAGCCCCACCATCTACATAGGTGAGTGCAGCTCAG
GCCTGTATGCTGTACCAGCGCTGGTGGACACCAACACGGCCACAATTTTCCACCGAGG
GTCACAGTTGCTGCTGGGGGGCCCTCCAGGTTCTCGAGGAACCATCAAGTCATCACAT
TCCCAAGCTGCCAGTGATGCAGAGAAAGAAAAGGAGCCTGAAGTTCTCCTGTTGGGT
TACTATGAAATACCGGAGAGTGCACAGACGGAGCTGCTCAAGGCCAAAACGGGGGAG
GTGACTGAGGGGAATGAGGAGGTGGTGCGTCTGGGCATCACTCACGCCAAGAACAAC
AGTCGGGCCTTGGAGGGGGAGGGACGGGGCGCCAGCAGGGACCAGCAGGACCTTGT
GGCTGTTGTCTGGGATGGAATGGAGCAAATCAAGATGATGAACATGAGTTTTGTGACC
TCTCAGAACATGTTTGTGTGGCTGGGGACCATTGGGCGCCTGGTGTGTGTCTGGCTGC
TGGACAACTACATGAACTTACTTGTGGTCACTCTTGCTTTTGGATTTTTAGCTATTTTAA
TTATTTTGTATAAGCAGGCGAAAGAATATGCACGGCTCAGTCGAGAAGTGTCCTCACG
GCGGCAGGGCTCCCAGGGCTCAACTGGTTCAGGCTCATCAAGCAGCGTCACAGCTGT
GGCGGAGGAGCTGAATGACGGCACCATTAGTGTGGGAAAGATCATTTTTAATCCACAA
AAGCTGCTTGGAAAGGGCTGTGATGGCACCTTTGTTTCAGAGGCAGTTTTGATGGCC
GGTCAGTGGCAGTAAAGCGAGTGCTCCCGGACTGCTTCTCTATTGCTGACCGAGAAGT

TGACCTGCTGCGGGAGAGTGACCAGCACCCCAACGTCATCAGATACTTCTGTACAGAG
CAGTGTCGTCAGTTTCGTTATATTGCCCTGGAGCTGTGCGGCGCCACTCTAGAGGACTT
CATACGGGGAAAATTCAAGGCAGACATCAGTATCCTGAGCATCCTTCATCAGGCCACCT
CAGGGCTTCATCATCTCCATCAGCTGGATATTGTTCACCGTGACATCAAGCCTCACAAT
GTGCTTCTTTCACTGCCAAACAACAAGGGACAAGTCAGGGCAATGTTGTCTGACTTTG
GGTTGTGTAAAAGGCTGGAGACAGGCCACATGTCCTTCTCCAGGAGGTCTGGGGTGA
CCGGCACGGAGGGGTGGATTGCCCCAGAGATGATGCTCAACACTTCTCGGCCAACATT
CAAGGTAGACATATTTTCTCTTGGTTGTGTGTACTACTACATGCTCACACAAGGAAAGC
ACCCGTTTGGTTCTGTCCTCGACCGGCAATCAAATATCATATCTGGAAAATTCAAACTG
GATGAGTTTGACAAAGAAAAGGATGTGAATTGCATCACCCTGATTGAGAGGATGGTGA
GCAGCAAGCCGTCGGAGCGGCCCTCAACATCTGCCATACTCAAGCACCCCTTCTACTG
GAACCCAGAGAAGGTTCTTGGGTTCTTCCAGGATGTGAGTGACCGTATTGAGAAGGAG
AACGGAGACACCATGGTTATGATGAGTCTGGAGTGGGGAGGAGCGCAGGTGGTGCAG
GAAGACTGGCGGCAGCACATGCACCCCGTCATTGCTGAAGACCTGCGCAGGTTCAGG
GACTACAAAGGACGCTCCGTGAGGGACCTCCTGCGAGCACTCAGGAACAAGCGGAAC
CACTACCGGGAGCTGAAGGAAGAGGCACGAATGGTGTTTGGCTCCATCCCAGACGAG
TTTGTGGCCTATTGGACCTTGAGGTTTCCCAGGTTGCTGCTACACACCTGGTGTGCCAT
GCAGTGTGTGAAGACGGAGGTCATATTCCAGAAGTACTATGACAAGAACTTCAACTTT
GGTCCGGACATAATTCCTGAGGCTCGTCAGTGTGCTCCAACCAGCACTCCCTGGCAGC
TGAGTGGCAACAGCCGAGTAGGGTCTACCTCCTCGGAGAGTCCATACCAGCGAGGAG
CAAACAAAACCTGCAAGGAGAAACAGGAAGTAAATCCTGTTTGGATTATTCGTAAATC
ACCCATAACTTCTGAACCCAAATATCGAATACCAATGCTAAGGAACATGGATGCTCCTT
GGAGGAGACCATTTGTGGAATCAAGCACAAAAGAAGTGAATCAGCATGAGACCGAAA
ATGATCCCCTACAGGGACAAGAAGCAGACATTTTAGGAAAAGAATTTAAGCTAGAAAA
TGCACTAGATATTGAGGAGTCCTCAATATCACTAGTTAGTGAGGCTCCAGAAGCAGAA
GCCTCTGTAGACCCAGAGAAGGTGAACCAAGAGTCCTCTCAACCACCTTGTGATGTGC
AGGATCAAGGCGAGACGAAATTCACTCATCCCTCCGAGGGTTCTGAAGGTGTAGATAA
AGAAGGTCCAGAAGAGCAGCCTGAGAATGGCAGGGAGGCATCCCGTCTGTGTATCAG
GGAGTCATCAAACGCCCAAGACAAGTCATCTAGTAAGCCAGACAGTGTTGTAAAGACC
GGAAAAGTCGCAGGGAAGAAGAAAAAATCTAAGAAATTACCTCTTGAGGAGACAAAC
AAATAG

Sequence No.9 *tfeb (partial)*
CAGGTGGAGGATGTCATCGACGAAATCCTTAGCCTGGAGGAGGGCTACGAGGCTGAC
ACTCGCAAGACCCCGGAGCAGCTGTGTGGCGTCCAGCCCGCGGCCAACAACAGTGTG
CCTGACCCACTATGCAATATACTGGGCCTCAACAGCTACATGGGCAAGACGTCCACCTC
GTGTCCTCCTGACGTGCCCCAGGTGAAGAGCGAGCCGCCCGTCAACGACCAGCAGCT
GGTGGCCTACATGAAGGACAGGCAGAAGAAGGACAATCATAATATGATTGAGCGAAGA
CGAAGGTTCAACATCAACGATCGCATCAAAGAGCTGGCCACTCTCCTCCCAAAGAGCA
ACGAACCGTACTTTGAACTCGTGCGAGACCTTCGCCACAACAAAGGTCAGATCCTCAA
GGCCTCCGTGGACTACATTCGGCGCCTCAAGCTGGACGCTGATGCCAACAAGGAAGC
GGAGGCCAAGCGTCGCGCCCTTGAGCAGCAGAACAGGCAGCTCCACCTTAGAATACA
GGAGCTGGAGGGAAGGCTGCGTTCAAATGGCATCAAGGTGGACGAGACCAACATAGA
TAAGAGTTTGCTCCTGTCAACGATCATTAAGACGGAGATG

Sequence No.10 *p62*

ATGCCGGAGGAGGCCAATATGAGTTTCAAGGTGTATCTGGACCTGGGTGGCGGCCGGC
AGGAGGTGCGGCGCTTTGCCCTGCCGGAGACTGTGGCCACCAACTACTCGGTGCTGA
GGGAGAAGGTCTGCAGTATCTTTGGCCTAGCGAACCAAGATGCCGTCCTCTCATGGAA
GGACTCCGAGGGTGATGCCATTGTCATCTCCACCGACGACGAACTGATGGAGGCCGTA
GCAGACAGCCTGAGCAACCAGAATGCCCAGTTGGTACGAATCAACGTGTCGGAGAGC
AGCGGCCGCCCAGAGCCTGGCACCGGCGGACGTCCAGGGAACCAGGTAGGAGACATC
CATCTTGGCGTGACCTGCGATGGCTGCAGTGGTGTCGTCCAGGGCTTCCGCTACCGCT
GCGTTGCCTGCCCGGACTACGACCTGTGCGGCGCCTGCGAGACCAAGGGAATGCATAA
GGAGCACAAGATGATCCGCCTGCCCAAGCCACAAGACCCTGGAGCTCCAAAGTTCTT
CTGGAAGGAGAGAGAGGAAGGGCCGGGCCAGTATTCAACTCACTTTGGGATGCAAGG
CGGTGATGCTGGTGGAGGTACTTTCCAGTCCAGCTCCTTCAGTGGCTGTGGTGCTGCT
GGTCCTGGGACTGGTGGAGGGGCTTGGTTTGGCTTTGGCGGTCCACGTGGGCGCTGCC
ACAGGCGGGGGCATGGAGGTGGTGGTCGGGGCCGTGGAGCCTGGCCAGGGTGGTGG
GGGCGAGGCTGGTCTGGGAAGAACTGGGCTGGCTGCGGTCAGGGGCAGACCATGGGC
TCAGACTCTGGATGTCAGCAACAGCAGCAGCAGCAGTCATCATCTGAGCAGCCCCAGC
AACAACAGTCCCAGCAGTCCCAGCAGCAACAGTCCCAGCAACAACAGAGTAATGACA
AGAGCCCCAAGGATGAGAGTGGGTGCCCTTGGCTGAATGCGGAGGGTTCTGCTTGCC
CCTTCATGGGTGAGGACACTGGTGTACCAACCCCGGAGCAGGTACAGCAGATGGCAC
AGCAGGCTGCTCATCAGGCCGCCCAGCAGGCCCACCACTTTGCCACGCACTACGCCTC
CGACCATCTCGCCAATGTCATGCGTGGAATCTGGACAGCTTGGTCTGGCCAGCAGGGA
CCGAACGCCACCACATCAACCTCCTCCTCCTCCTCCAGCTCCTCCTCCTCCCACTC
TGCCAGTGCCAGCCAGGACGGCCCCAAGCAGGGTACAGCAGAGGAGCAGAAGAGCG
AGCAGGGAGGGCAGTTTGTCAGGGAGGAGTACCTGAGGAACGTTGGGGAGACGGTT
GCCAACATGCTGGACCCTCTCGGCATTGACGTGGAGGTGGCCGTGGAGCACAATGGG
ATTCGCCAGCGCTGCAGCCTCGGGGAAAACGCACCCACCTCACAGAGCAACGCCTCA
CCAACCCCAACCAGCCCCACCAAGCAACCAGAGTCCACACCTTCCTCCTTGGCCTCCG
GAAGCGTGAACATGGAGGTGCAGACCGAGGAACCTGCAGAACCACAGACGCCAAGT
ACCAAGGATGTCGAGGCTATGGATGTCGCAAGCCCAGCACCAGCCCCAGTTAGCCAGG
TCGCAGGGGGGCAGGCAGAGGGTCACACTGGGTCCCCTGAACCCGAGGACTGGATGC
TAGTGAATCAGGAGACGAGTGAGCCTGCCTCAGCCTCCGGGGTCAGACCCAAGACCC
CACCAAATGATAAGCCAGTGTCCTACCCAAGTCTGAAGGAGCTCACGCCTCATCCTGA
CCCCAAGATCCAGCAGTCCTTGGAGCAGATGTCAGCGATGGGCTACAGCAACGAGGG
AGGCTGGCTGACCAACCTGCTGGAGATGAAGCAGGGAGACATCAACCAAGTCCTGGA
CCTCCTGCAGCCGGTCAATAAGTAA

Sequence No.11 *lc3c*

ATGAGGCCCCGACCACGTGTGATGAGGTGCAGCTGTGTGTTCTGTGCTAAAGCCCCGG
ACAGCAGTACAAGCATTAGTAAGGACTGCGAGACTGCTTATTTCCCCTACCTCGGCGCT
GGCATTGCAGGACCCGTAGTGGTGCCCAAAACCTTCAAGCAGATGTACAGTTACACCT
CCAGACAACAGGAAGTGGCGAATATCAGAAGTAAATTCCCGAACAAAATTCCCGTGGT
GGTGGAGAGGTACGCGAAGGAGACCACGTTGCCTATCCTCGACAAGACCAAGTTTCT
GGTGCCTCAGGAGCTCACCATGTCCCAGTTCGTCACCATTCTCAGGAACAGAATGCAG

CTCCAATCCACCATGAGTTTCTACCTCCTCGTCAACAACCGCTCGCTGGTCTCCCTCTC
GCGGCCGCTCTCCGAGGTGTACCGCGAGTGTAGGGACGATGACGGCTTCCTCTACGTC
ACCTACGCCAGCCAGGAGGTCTTCGGCTGAGGGCAGGACAGATGAGGTCCGGTCAAG
GTGCTCAAGCTTTGTACTGCTGTGATAG

Sequence No.12 *lc3a*
ATGAATGCACAGGTTAAGCCCTTCAGGGAGCGACGGAGCTTTGCCCAGAGGCAGCGT
GACGTGGAGCAGATCCGCGAGCAGCACCCCAACAAGGTCCCGGTGATCATCGAGCGT
TACCCCGGCGAGCGCCACCTCCCACTGCTGGACAAGACCAAGTTCCTGGTGCCTGACC
ACGTCACGATGGGAGAACTGGTCAAGATTATTCGCCGTCGCCTCCAGCTGCACCCGAC
ACAGGCTTTCTTCCTTCTGATCAACCAACGCTCTCTTGCCAATGTGTCCAGCACCCTCG
CCCAGGTTTACGAGCACCACCACCACGAGGACGGCTTCCTCTACATGGTGTATGCGTC
CCAGGAGGTGTTTGGATAA

Sequence No.13 lamp (partial)
ATCAAGTACAGCGTGACTGATGAAAATAATGTGACCTGCATTATTGTAGCAGGGGAAAT
ATTTTTCAATGTCAACTACACACTCCGTGACAATACAACTGCAGTCAAGTCTGTGTTTG
TGCCATCCATTGGTGTCACAGTTGATGGGTCTTGTAATACAACTGAAGGCAATCAGTTC
ATCTCATTCTCTTGGGACAAAAACAACTCTGTCCAGATGAACTTTGAAATTGGAGACA
AGGATGCCTGGATGTTCACAACCCTCACAGCCTCCCTGCTCATGGATGATGACTCATTT
GCAAATGCTACAGATGCTGGCAAGACACTTGACCTGACAGTAGATTACAACTTTAGCC
CTGGTGAAGTGAGTGTCAACCACAGCTTCATCTGCCGCTCCTCCATTACCACCAACAAT
GTGACTGCCACCATTGAAAAGGATACTTACAGCTCTCCAGTCACATCTACCCTGAAGA
ACATCCAAATGCAGGCCTTCAACAAGATAAAGGGTCAGGAAAACTTTGCAGAGTCTGT
ACACTGCACTGCCGACAACACCTCGGATGTGGTACCCATTGCTGTTGGCTGTGCTCTG
GCTGGCCTGGTAGTGATTGTGCTCATTGCCTATCTGGTAGGTCGTCGCAAAAGGTCGGG
AGCATACCAGTCGGTG
